# Many *Saccharomyces cerevisiae* Cell Wall Protein Encoding Genes Are Coregulated by Mss11, but Cellular Adhesion Phenotypes Appear Only Flo Protein Dependent

**DOI:** 10.1534/g3.111.001644

**Published:** 2012-01-01

**Authors:** Michael C. Bester, Dan Jacobson, Florian F. Bauer

**Affiliations:** Institute for Wine Biotechnology, Faculty of Agricultural and Forestry Sciences, Stellenbosch University, 7600 Stellenbosch, South Africa

**Keywords:** Mss11, *FLO*, cellular adhesion, cell wall

## Abstract

The outer cell wall of the yeast *Saccharomyces cerevisiae* serves as the interface with the surrounding environment and directly affects cell−cell and cell−surface interactions. Many of these interactions are facilitated by specific adhesins that belong to the Flo protein family. Flo mannoproteins have been implicated in phenotypes such as flocculation, substrate adhesion, biofilm formation, and pseudohyphal growth. Genetic data strongly suggest that individual Flo proteins are responsible for many specific cellular adhesion phenotypes. However, it remains unclear whether such phenotypes are determined solely by the nature of the expressed *FLO* genes or rather as the result of a combination of *FLO* gene expression and other cell wall properties and cell wall proteins. Mss11 has been shown to be a central element of *FLO1* and *FLO11* gene regulation and acts together with the cAMP-PKA-dependent transcription factor Flo8. Here we use genome-wide transcription analysis to identify genes that are directly or indirectly regulated by Mss11. Interestingly, many of these genes encode cell wall mannoproteins, in particular, members of the *TIR* and *DAN* families. To examine whether these genes play a role in the adhesion properties associated with Mss11 expression, we assessed deletion mutants of these genes in wild-type and *flo11*Δ genetic backgrounds. This analysis shows that only *FLO* genes, in particular *FLO1*/*10*/*11*, appear to significantly impact on such phenotypes. Thus adhesion-related phenotypes are primarily dependent on the balance of *FLO* gene expression.

Yeast cells are enclosed by a rigid but dynamic cell wall structure that forms a physical barrier to the extracellular environment. The cell wall is composed of interlinked β-glucan polysaccharides and, to a lesser extent, chitin, and acts as the supporting scaffold for highly glycosylated mannoproteins. Mannoproteins are polypeptides that are extensively modified by covalently bound branched polymers of mannose residues (Lesage and Bussey 2006), and define the characteristics of the outer physical profile of yeast cells. One family of cell wall proteins, referred to as Flo proteins or yeast adhesins, has been shown to function in cell−cell as well as cell−substrate recognition and adhesion (Dranginis *et al.* 2007). Adhesin-mediated phenotypes include flocculation (Guo *et al.* 2000; Verstrepen and Klis 2006), agar adhesion and/or invasion (Guo *et al.* 2000; Verstrepen and Klis 2006), the formation of pseudohyphae (Lambrechts *et al.* 1996; Lo and Dranginis 1996; Lo and Dranginis 1998) or biofilms (Purevdorj-Gage *et al.* 2007; Reynolds and Fink 2001), the adherence to plastic surfaces (Mortensen *et al.* 2007), colony morphology (Kuthan *et al.* 2003), as well as “flor”/“velum” formation that occurs during the ageing of sherry (Fidalgo *et al.* 2006; Ishigami *et al.* 2006). Adhesin-encoding genes typically contain internal tandem repeats that may expand or contract by means of recombination (Verstrepen *et al.* 2005). Verstrepen *et al.* (2005) showed that an increase in repeat length can be directly correlated with the increase in Flo1-dependent phenotypes such as flocculation and plastic adherence. *FLO* gene expression has also been correlated with changes in the general physical-chemical properties of the cell wall. For instance, the overexpression of individual *FLO* gene family members strongly and differentially impacts on cell wall hydrophobicity (Govender *et al.* 2008, 2010).

However, information regarding the regulation of genes responsible for cell-wall dependent phenotypes remains limited, with the exception of *FLO11* and to a lesser degree *FLO1* (Chen and Thorner 2007). *FLO11* is the only nonsubtelomeric *FLO* family member and thus is not subjected to telomere silencing. However, the gene has been shown to be under epigenetic control (Halme *et al.* 2004; Octavio *et al.* 2009). Flo11 is required for and/or contributes to the formation of pseudohyphae (Lambrechts *et al.* 1996; Lo and Dranginis 1996, 1998), “flor” formation (Ishigami *et al.* 2006), “mat” formation (also referred to as “biofilm formation” or “yeast sliding motility”) (Reynolds and Fink 2001), as well as flocculation in *S. cerevisiae* var. *diastaticus* (Bayly *et al.* 2005). Although increased expression of other adhesin encoding genes can compensate for the absence of Flo11, as has been shown for Figure 2 and *FLO10*, whose overexpression can support pseudohyphal development in yeast carrying a *FLO11* deletion (Guo *et al.* 2000), the biological relevance of such artificially generated phenotypes remains uncertain. *FLO1* encodes a dominant flocculation factor and appears to be exclusively required for cell-cell adhesion (Goossens and Willaert 2010; Goossens *et al.* 2011). Overexpression of the *FLO1* homologs *FLO5* and *FLO9* induces flocculation of a broadly similar nature to that of *FLO1* but at different levels of intensity that appears largely strain-dependent (Govender *et al.* 2008). Although the expression of specific adhesins, or alleles thereof, leads to different phenotypic outcomes, it remains unclear how adhesion-encoding genes are differentially regulated to facilitate specific phenotypic outcomes that would be appropriate in specific environmental conditions. Importantly, it also remains to be clarified whether the expression of other proteins that may be coregulated with the adhesins is contributing to specific cell wall−related phenotypes.

Mss11 performs a central role in the regulatory mechanisms by controlling *FLO11* and *FLO1* expression (Bester *et al.* 2006; Van Dyk *et al.* 2005), and Flo11-dependent phenotypes are all Mss11-dependent (Barrales *et al.* 2008; Gagiano *et al.* 1999b). Here, we identify other genes whose transcription is significantly altered in strains overexpressing or carrying deletions of *MSS11*. For this purpose, two commonly used and phenotypically diverging laboratory strains, S288c and Σ1278b, were investigated by means of whole transcriptome analysis. Σ1278b is generally used to study the formation of pseudohyphae and the ability of yeast to grow invasively into agar containing media. S288c, on the other hand, is the most commonly used laboratory yeast but is unable to form pseudohyphae or grow invasively because of a nonsense point mutation (*flo8-1*) in another transcriptional activator of *FLO1* and *FLO11*, Flo8 (Liu *et al.* 1996). Restoration of the genomic copy of *FLO8* leads to the reestablishment of both flocculation and invasive growth in this strain (Bester *et al.* 2006; Liu *et al.* 1996).

Our analysis shows that most of the genes identified as being strongly affected by changed concentrations of Mss11 in both S288c and Σ1278b genetic backgrounds encode cell wall mannoproteins, suggesting that Mss11 is primarily involved in the modulation of cell wall properties. However, a genetic analysis suggests that none of these genes appears to contribute to the phenotypes that depend on *FLO* gene expression. Furthermore, our data show that some of the genes that are up-regulated in response to increased expression of *MSS11* in fact respond to the increased expression of *FLO11* observed in such strains and are probably not direct targets of Mss11.

## Materials and Methods

### Plasmids, strains, media, and culture conditions

Plasmids and *S. cerevisiae* strains used in this study are listed in supporting information, Table S1 and Table S2, respectively. All strains are isogenic to either the S288c or Σ1278b genetic backgrounds. *FLO8* replacement in strains carrying the *flo8-1* allele was performed as described previously (Bester *et al.* 2006). By using genomic DNA isolated from the corresponding European *Saccharomyces cerevisiae* Archive for Functional Analysis (EUROSCARF) gene deletion library strains as a template, we amplified gene deletion cassettes containing the *KanMX4* selection marker via polymerase chain reaction (PCR) with the primers listed in Table S3. These deletion cassettes were subsequently used to generate deletions in the BY4742 *flo8-1*Δ::*FLO8-LEU* and Σ1278b genetic backgrounds. Yeast transformations were performed according to the lithium acetate method (Ausubel 2004). Yeast cultures were grown at 30° except for the assessment of “mat” formation (see *Mat formation*). Yeast peptone dextrose (YPD) was used as rich media. Minimal media contained 0.67% yeast nitrogen base with preadded ammonium sulfate but without amino acids supplemented with 2% glucose (w/v) and the required amino acids (SCD media) according to the auxotrophic growth requirements of the relevant strain. Low nitrogen (SLAD) media was prepared similar to SCD except that 0.17% yeast nitrogen base without amino acids or ammonium sulfate was used with the addition of ammonium sulfate to a final concentration of 50 μM. Selection for the *KanMX4* marker was performed by supplementing media with 200 mg/L Geneticin (G418; Sigma-Aldrich, South Africa).

### Preparation of yeast total RNA

Yeast cultures were grown in 5 ml of SCD media from an optical density of 0.1 to between 1 and 2 as determined by spectrophotometric absorbance at a wavelength of 600 nm. Cells were harvested, washed with H_2_O, and resuspended in an ice-cold buffer containing 50 mM sodium acetate, 10 mM EDTA at a pH of 5.0. Total RNA was extracted as described previously (Schmitt *et al.* 1990). For transcript analysis, total RNA from three independent biological repeats was analyzed.

### Microarray analysis, data normalization, and differential expression

Probe preparation and hybridization to Genechip microarrays (Affymetrix; Santa Clara, CA) were performed according to Affymetrix instructions, starting with 6 μg of total RNA extracts. Results for each strain were derived from three independent culture replicates. Quality of total RNA, cDNA, cRNA, and fragmented cRNA were analyzed by use of the Agilent Bioanalyzer 2100. Probe hybridization to GeneChip Yeast Genome 2.0 Arrays was performed on the integrated Affymetrix GeneChip 3000 platform. Chip scanning and data collection were performed with the Affymetrix GeneChip Operating Software (GCOS) version 1.4. (http://www.affymetrix.com/support/technical/manuels.affx). Data sets are available from the Gene Expression Omnibus web site under the series records GSE17716 and GSE29371. The microarray data were background corrected and normalized with RMA (Irizarry *et al.* 2003) and the resultant log_2_ transformed intensity values compared for each of the overexpression or deletion strains with their respective wild-type strains. Determination of differential gene expression between strains was conducted by the creation of an R script by use of the linear fitting and empirical Bayes methods of limma (Smyth 2004, 2005). Significant differential gene expression was determined by the use of a threshold for a Benjamini and Hochberg corrected *P* value < 0.05. Differential expression is reported as log fold changes.

### Gene ontology enrichment analysis

Differentially expressed probe sets were analyzed for Enrichment of Gene Ontology Terms by GOEast with default settings (Zheng and Wang 2008), including a calculated (Benjamini and Yekutieli 2001) FDR threshold of 0.1.

### Quantitative real-time PCR (qPCR) analysis

DNA contamination in total RNA samples was eliminated by DNase I (Roche Diagnostics, Indianapolis, IN) treatment. One microgram of total RNA was used as template for cDNA synthesis with the ImProm-II reverse transcription system according to the manufacturer instructions (Promega, Madison, WI). cDNA samples were diluted 50 times with H_2_O before qPCR analysis. Primers and hydrolysis probes used for detection and quantification of cDNA were designed with Primer Express ver. 3 (Applied Biosystems, Carlsbad, CA) and are listed in Table S4. Detection reagents were purchased from Applied Biosystems and Kapa Biosystems (Cape Town, South Africa). qPCR runs and collection of spectral data were performed with a 7500 cycler (Applied Biosystems). Except for cDNA corresponding to transcripts of *FLO1*, *FLO5*, and *FLO9*, amplicon formation was monitored with SYBR Green fluorescence with individual primer concentration of 100 nM. Specific labeled hydrolysis probes (Taqman) and primers were designed to differentiate between the cDNA species corresponding to the highly homologous *FLO1*, *FLO5*, and *FLO9* genes. Hydrolysis probes were modified by the addition of a 3′ minor groove binding protein and nonfluorescent quencher, as well as the 5′ attachment of fluorescent dyes as described before for the *FLO1* and *FLO5* specific hydrolysis probes and primer sets (Govender *et al.* 2008).

The hydrolysis probe (Applied Biosystems) and primer set used for *FLO9* cDNA detection are listed in Table S4. Hydrolysis probe and primer concentrations were kept at 250 nM and 900 nM, respectively, for reactions containing probe primer combinations. Cycling conditions during qPCR were as follows: 50° for 2 min, 95° for 10 min, 40 cycles of 95° for 15 sec, followed by 60° for 1 min. When we used SYBR Green for amplicon quantification, a dissociation curve analysis was included after the cycling program to verify amplicon authenticity. Preliminary data analyses were performed with Signal Detection Software, ver 1.3.1. (Applied Biosystems). Individual qPCR reaction runs were performed at least in duplicate. The relative expression value for each sample was defined as 2^-Ct^_(target)_, where Ct_(target)_ represents the cycle number at which a sample reaches a predetermined threshold signal value for the specific target gene. Relative expression data were normalized to the relative expression value of the reference gene *PDA1* (Wenzel *et al.* 1995) in each respective sample, thus giving normalized relative expression for a target gene as 2^-Ct^_(target)_/2^-Ct^_(_*_PDA1_*_)_. Fold change was calculated by log_2_-converting the data followed by subtracting the value for the reference condition/strain.

### Flocculation and hydrophobicity assay

Ca^2+^-dependent flocculation of yeast cultures was determined by a method based on the Helm’s sedimentation test (Bester *et al.* 2006). Yeast hydrophobicity was measured by assaying the partitioning of yeast cells between an aqueous and hydrophobic hydrocarbon phase after vigorous mixing (Rosenberg 2006). Yeast cultures were deflocculated by the addition of ethylene diamine tetra-acetic acid (EDTA), after which the spectrophotometric absorbance was determined at a wavelength of 600 nm (measurement A) as described in the flocculation protocol. A total of 1 ml of yeast culture was transferred to a microcentrifuge tube, washed, and resuspended in phosphate, urea, magnesium buffer consisting of 127.45 mM K_2_HPO_4_, 53.35 mM KH_2_PO_4_, 30 mM urea, and 0.8 mM MgSO_4_ (Hinchcliffe *et al.* 1985). Finally 100 μl of *p*-Xylene (1,4-dimethylbenzene) was added. Samples were vortex-mixed vigorously for 30 s and left to stand for 15 min, whereupon the spectrophotometric absorbance of the aqueous phase was determined at a wavelength of 600 nm (measurement B). The hydrophobicity index (HI) was defined as 1 − (B/A), where greater values reflect a yeast population of an increased hydrophobic nature.

### Invasive growth determination

To investigate the ability of yeast cultures to grow invasively into agar-containing medium, 10 μl of yeast suspensions grown overnight to stationary phase were deposited on 2% agar plates with various media composition as indicated for each specific experiment. Flocs in flocculating cultures were disrupted by repetitive pipetting, and a sample was immediately removed and the OD_600_ determined as described previously. Cultures were adjusted so as to contain the same concentration of cells, washed with water, and spotted on plates. Spotted macrocolonies from flocculating cultures have a granular appearance because of cells that reform flocs on the plate after spotting. After allowing for yeast growth at 30° for times depending on specific experiments as indicated, we washed cells off of the agar surface by vigorous rubbing with a gloved finger under running water, revealing only those cells that have grown into the medium.

### Mat formation

The ability of yeast strains to form spreading growth mats (also referred to as “biofilm” formation or “sliding motility”) on plates was determined as described previously (Reynolds and Fink 2001). In brief, 10 μl of a yeast suspension grown overnight in liquid media as described previously was deposited in the center of an YPD plate containing 0.3% w/v agar and incubated at room temperature (20−25°). “Mat” formation was monitored by measuring the diameter of growth of at least three independent biological repeats. Measurements were always taken by use of the same reference point on the plate.

### Polystyrene adherence assay

To measure the ability of yeast cells to adhere to polystyrene plastic surfaces, liquid cultures (100 μl) were incubated at room temperature in flat bottom polystyrene 96-well plates (Sterilin). After incubation (~2 hr) an equal volume of a solution of 1% (w/v) crystal violet was added to the cells followed by further incubation for 15 min at room temperature. The wells were repeatedly washed with H_2_O, leaving only stained cells remaining attached inside the wells. Sodium dodecyl sulfate was added to the wells to desorb the crystal violet from the cells and increase the visibility of attachment (Reynolds and Fink 2001).

## Results

### Altered *MSS11* expression affects transcription in S288c and Σ1278b

To discover novel targets of Mss11, genome-wide expression levels of strains with modified *MSS11* expression were monitored through DNA microarray analysis. These strains included the Σ1278b and S288C wild-type strains transformed with the multicopy shuttle vector YEpLac195 containing *MSS11* (2μ-*MSS11*) as well as the Σ1278b *MSS11* deletion strain (*mss11*). The same strains transformed with the YEpLac195 (2μ) without insert were used as controls. Total RNA for transcriptome analysis was isolated from three biological replications of transformants grown to the mid-exponential growth phase.

As expected no *MSS11* transcript was detected in Σ1278b *mss11*Δ::*LEU2* (data not shown), whereas strains with 2μ-*MSS11* displayed a 3.2- and 4.3-fold up-regulation in S288c and Σ1278b respectively. This finding is in agreement with previous findings showing that this multicopy expression system results in the up-regulation of Mss11-specific targets such as *FLO1* and *FLO11* (Bester *et al.* 2006; Gagiano *et al.* 2003; Van Dyk *et al.* 2005).

Listed in Table S5 are the genes found to have statistically significant (see *Materials and Methods*) change in their expression profiles for each of the three strains when compared with the corresponding wild type. A total of 77 genes were significantly affected (20 down-, 57 up-regulated) in S288c overexpressing *MSS11* whereas three times less genes were affected in Σ1278b (total: 26; 2 down-, 24 up-regulated). *MSS11* deletion in Σ1278b resulted in 7 down-regulated genes. A Gene Ontology (GO) enrichment analysis (Table S6) was performed, and most genes were grouped in cell wall related categories, including “anchored to membrane” (GO:0031225), “cell periphery” (GO:0071944), “cell wall” (GO:0005618), “external encapsulating structure” (GO:0030312), “extra cellular region” (GO:0005576), “filamentous growth” (GO:0030447), “flocculation” (GO:0000128), “fungal-type cell wall” (GO:0009277), “intrinsic to membrane” (GO:0031224), “multi-organism process” (GO:0051704), and “plasma membrane” (GO:0005886). These categories were found to typically contain the same set of genes belonging to two protein families: (1) *DAN1*, *TIR1*, *TIR2*, *TIR3*, and *TIR4* from the Srp1/Tip1 family reported to respond to hypoxia and cold stress (Abramova *et al.* 2001a, 2001b; Sertil *et al.* 1997; Tai *et al.* 2005; Ter Linde *et al.* 1999) and (2) *FLO1*, *FLO5*, *FLO9*, and *FLO11* from the flocculation (*FLO*) family encoding for cell wall adhesins (Soares 2011).

**Figure 1:**
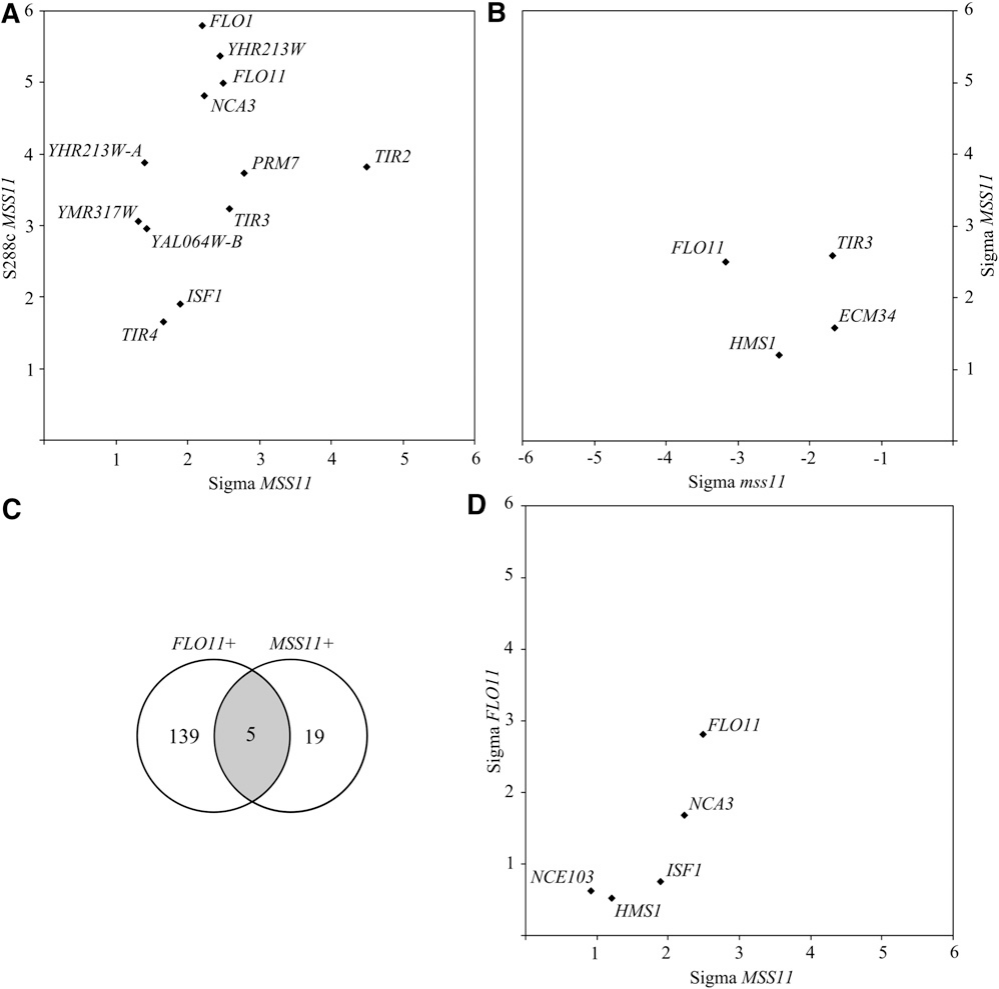
Genes significantly regulated in both of the following strain comparisons. (A) The *MSS11* overexpression strains S288c (S288c *MSS11*; y-axis) and Σ1278b (Sigma *MSS11*; x-axis), (B) *MSS11* overexpression and deletion in Σ1278b (Sigma *MSS11* and Sigma *mss11* on the y- and x-axis respectively), (C) Venn diagram depicting the amount of regulated genes either shared or unique across the overexpression strains, and (D) Σ1278b either overexpressing *FLO11* (Sigma *FLO11*; y-axis) or *MSS11* (Sigma *MSS11*; x-axis).

Twelve genes were up-regulated in both S288c and Σ1278b, including *TIR2*/3/4 and *FLO1*/*11* (Figure 1A). Uncharacterized genes regulated in this manner are *YMR317W*, the pseudogene *YHR213W* (with *FLO1* sequence similarity) (Teunissen and Steensma 1995), *YHR213W-A* (located adjacent to *YHR213W*), and the “fungal-specific” *YAL064W-B* (Nishida 2006). The remaining genes do not have cell wall−related function: *ISF1* is involved in mitochondrial function (Altamura *et al.* 1994), *PRM7* is responsive to pheromones (Heiman and Walter 2000), and *NCA3* encodes a transcriptional regulator of subunits 6 (Atp6) and 8 (Atp8) of the mitochondrial Fo-F1 ATP synthase (Pelissier *et al.* 1995).

In Σ1278b *ECM34*, *FLO11*, *HMS1*, and *TIR3* were found to be induced upon *MSS11* overexpression and repressed in an *MSS11* deletion strain (Figure 1B). *HMS1* was previously identified as a regulator of pseudohyphae formation (Lorenz and Heitman 1998), and *ECM34* is an uncharacterized gene with suspected cell wall function (Lussier *et al.* 1997).

### Common gene targets of both *FLO11* and *MSS11* overexpression

To determine to what extent high levels of Flo11 may account for the effects observed in the *MSS11* overexpressing strains, we performed a microarray analysis of strain Σ1278b overexpressing *FLO11* by using the constitutive strong promoter from *PGK1* and with wild-type Σ1278b as reference. The experimental conditions were similar as for the *MSS11* overexpression analysis, and in this manner 139 genes were found to be significantly regulated in response to *FLO11* overexpression. GO enrichment analysis (Table S7) indicates that these genes are predominantly involved in metabolic functions. Five of these genes were also and similarly affected by upon *MSS11* overexpression (Figure 1, C and D) and are involved in metabolic or mitochondrial functions. These genes are *NCA3*, *HMS1*, *ISF1*, *NCE103*, and as expected *FLO11. NCE103* encodes the only carbonic anhydrase for yeast (Amoroso *et al.* 2005). The *MSS11* and *FLO11* data sets display unique levels of variation across their respective biological repeats. As a consequence, more genes appear to be affected in a statistically significant way in the *FLO11* overexpressing strain than in the MSS11 overexpressing strain, although *FLO11* levels are similarly up-regulated in both strains.

### qPCR gene expression analysis

**Figure 2:**
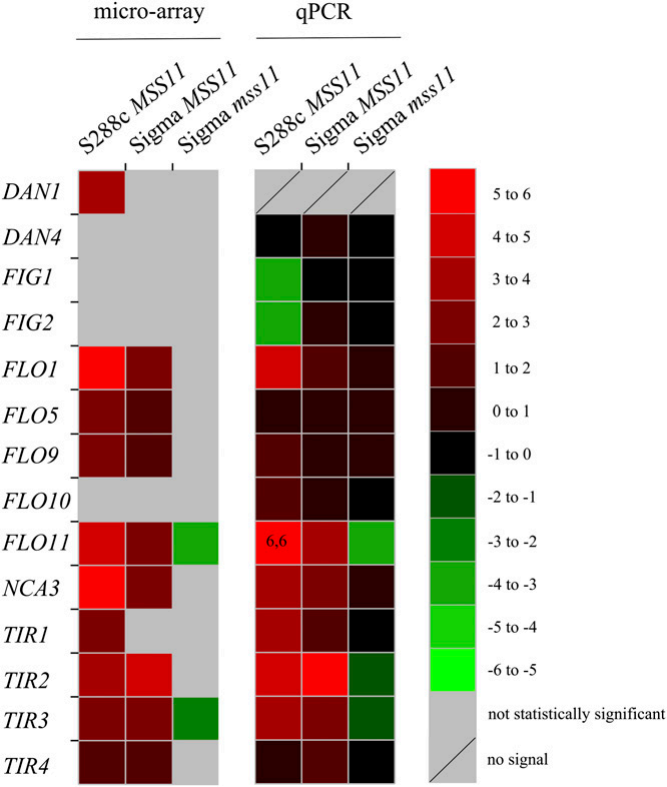
*MSS11* deletion and overexpression regulates a selection of cell wall−associated genes. Expression fold change (log_2_ transformed) is indicated in the scale with red representing up-regulation and green down-regulation, respectively. Fold changes falling outside the range of the indicated scale is represented as a numerical value displayed on a saturated color background. As indicated, the left and right panels represent data from the microarray and qPCR analyses respectively. Only microarray data with significant fold changes are shown (see *Materials and Methods*). No signal was detected for *DAN1* in the reference strain as analyzed with qPCR. Color map generated by JColorGrid ver 1.860 (Joachimiak *et al.* 2006).

To confirm the data, *DAN1*, *FLO1/5/9/11*, *and TIR1/2/3/4* were analyzed by means of quantitative real-time PCR (qPCR). In addition *DAN4*, *FIG1*, *FIG2*, and *FLO10* were also included in this analysis to explore potential functions of other members of these gene families.

qPCR analysis shows that *FLO5* and *FLO9* are not regulated as the microarray analysis suggests (Figure 2). This difference is likely because of the nature of the Affymetrix Genechip probe sets, which cannot efficiently differentiate between *FLO1*, *FLO5*, and *FLO9* signals because of very high sequence homologies. *FLO1*, *FLO11*, and *NCA3* up-regulation in the *MSS11* overexpression S288c and Σ1278b strains are confirmed, as well as *FLO11* repression in response to *MSS11* deletion. *FLO10* and all *TIR* members follow the same expression pattern as *FLO11*, albeit with lower magnitude. As for *DAN1*, a signal with substantial variation between biological repeats, was detected in overexpression strains but not in the reference or deletion strain (Figure S1), suggesting that *MSS11* overexpression does impact on this gene. The data furthermore show that *DAN4* is induced in the Σ1278b *MSS11* overexpressing strain.

In the S288c genetic background, *FIG1* and *FIG2* are the only genes down-regulated in response to *MSS11* overexpression. Both of these genes are important for mating (Aguilar *et al.* 2007; Erdman *et al.* 1998; Muller *et al.* 2003; Zhang *et al.* 2002), suggesting a possible function of Mss11 in reducing mating while increasing other adhesion-related phenotypes.

**Figure 3:**
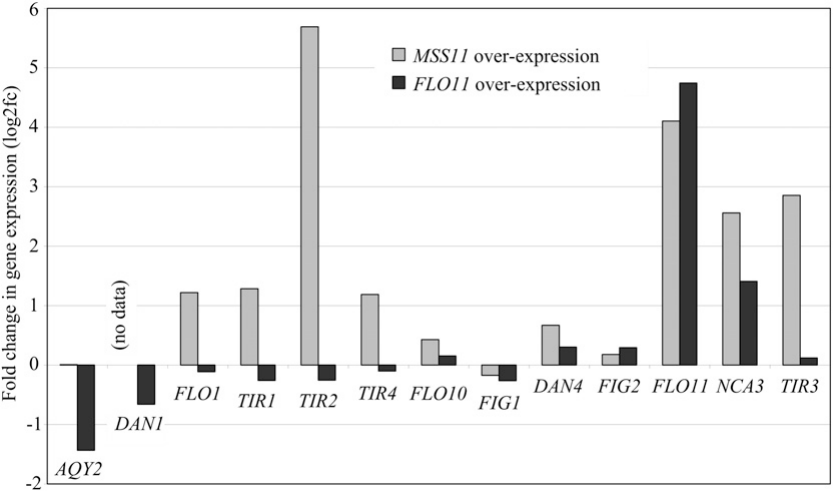
Fold change of expression of selected genes in response to the overexpression of either *MSS11* or *FLO11* in strain Σ1278b.

We further extended the qPCR analysis to compare the *MSS11* overexpressing strain with a Σ1278b strain constitutively overexpressing *FLO11* (Figure 3). *FLO11* is induced to a very similar degree (4.7- and 4.1-fold) in the *FLO11* and the *MSS11* overexpressing strains, but none of the cell wall−associated genes included in this analysis was significantly affected in the strain overexpressing *FLO11*.

*NCA3*, the only non-cell wall protein assessed here, is strongly induced in response to high levels of *FLO11* expression, similar to what is observed in the Mss11 overexpressing strain. We also included *AQY2* in the analysis because it has been shown to be regulated in a manner similar to *FLO11* (Furukawa *et al.* 2009), but our data suggest a 1.4-fold reduction in the expression of this gene in response to *FLO11* overexpression.

### Adhesion phenotypes of transformants

**Figure 4:**
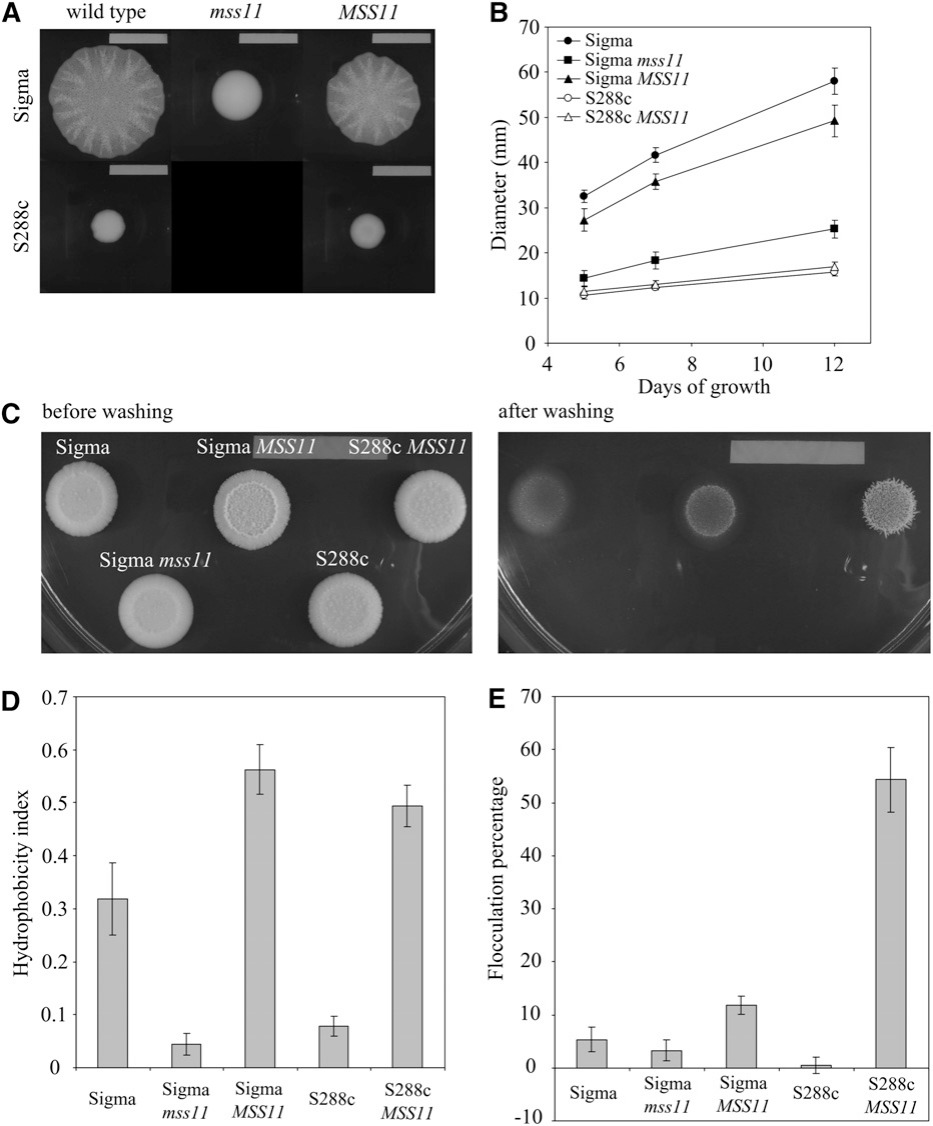
Adhesion phenotype analysis of the Σ1278b (labeled Sigma) and S288c transformants used in the transcriptome analysis. Wild-type, *MSS11* deletion (*mss11*), and overexpression (*MSS11*) strains were analyzed as indicated. (A) “Mat” formation on 0.3% YPD agar after 7 days of growth. (B) Measurement of “Mat” growth at day 5, 7, and 12, respectively. (C) Invasive growth of transformants. Transformants were grown in selective media and spotted on YPD plates: Total growth after 6 days incubation (left) and invaded cells revealed following subsequent plate washing (right). Transformants grown to stationary phase in liquid minimal media (SCD) assayed for (D) the degree of culture hydrophobicity and (E) their ability to flocculate.

Phenotypes dependent on cell wall adhesins were assessed for Σ1278b, S288c, and S288c carrying a reconstituted copy of *FLO8* (see Figure S2), as well as for the transformants used for the transcriptome analysis (Figure 4). Phenotypes monitored included “mat” formation (Reynolds and Fink 2001) (Figure 4, A and B, Figure S2, A and B), adherence to wells of polystyrene plates (Figure S2C), agar invasion (Figure 4C), cell wall hydrophobicity (Figures 4D and Figure S2D), and flocculation (Figures 4E and Figure S2E). Although S288c wild-type is unable to undergo “mat” formation or flocculate, these phenotypes are restored in the strain with a functional copy of *FLO8*. “Mat” formation by S288c (*FLO8*) appears, however, uniquely different from that formed by Σ1278b, and the strain only forms fully developed growth “mats” after an extended incubation of 3 weeks with extensive morphological variation between biological repeats. In addition, *FLO8* replacement increases cell wall hydrophobicity and ability to adhere to polystyrene surfaces. Irrespective of genetic background, an abolishment of all the aforementioned phenotypes is observed upon *MSS11* deletion.

In accordance with previous reports (Barrales *et al.* 2008; Bester *et al.* 2006; Gagiano *et al.* 1999b), Mss11 was shown to be absolutely required for flocculation and agar invasion and affects cell wall hydrophobicity. We further show that Mss11 is required for “mat” formation as strain Σ1278b *mss11*Δ is unable to form this specific growth form as displayed by wild type (Figure 4, A and B). The same deletion strain cannot invade agar plates (Figure 4C) and shows a decrease in cell wall hydrophobicity (Figure 4D). Remarkably, Σ1278b displays a low level of flocculation (~5%) under these growth conditions (Figure 4E). *MSS11* overexpression restores invasive capability and flocculation in S288c and leads to increased invasion and floc formation (~12%) in Σ1278b. Both *MSS11* overexpressing strains display increased cell hydrophobicity. Interestingly, *MSS11* overexpression could not suppress *flo8-1* in S288c with regard to the lack of “mat” formation, even after extending the incubation period (data not shown). Furthermore *MSS11* overexpression led to “mats” of a smaller diameter in comparison to wild type Σ1278b. The overexpression of either *FLO11* or *MSS11* in Σ1278b results in an increase of invasion, cell wall hydrophobicity, and low levels of flocculation (Figure S3). Thus both strategies result in similar adhesion phenotypes.

### Adhesion phenotype screen of single- and their corresponding *flo11*Δ double-deletion strains

To assess possible roles of the other cell-wall protein encoding genes that our data show are coregulated with *FLO* genes, double-deletion strains were constructed in which the deletion of *FLO11* was combined with deletions of each of the investigated genes, *DAN1*, *FIG1*, *FIG2*, *FLO1*, *FLO10*, *NCA3*, *TIR1*, *TIR2*, *TIR3*, or *TIR4* in both the Σ1278b and S288c (*FLO8*) genetic backgrounds. These double-deletion strains (and the *FLO11* single-deletion strain as control) were furthermore transformed with either an empty vector or 2μ-*MSS11* and spotted on low nitrogen media (SLAD) to investigate invasion.

**Figure 5:**
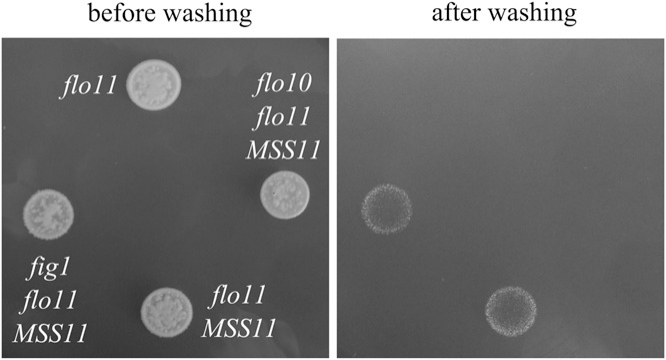
Invasion of *flo11*Δ single and double mutants: Total growth after 6 days on SLAD plates (left) and cells that invaded the agar medium revealed by washing the plate (right). Strain S288c (*FLO8*) *flo11*Δ *fig1*Δ transformed with 2μ-*MSS11* is representative of all of the deletion strains transformed with the same construct with regards to agar invasion with the exception of strain S288c (*FLO8*) *flo11*Δ *flo10*Δ.

We previously reported that overexpression of *MSS11* in a Σ1278b *flo11*Δ strain led to the reestablishment of some invasive growth. This phenotype was not suppressed in any of the double mutants (Figure 5). However in S288C (*FLO8*) *flo11*Δ, the deletion of *FLO10* was found to abolish agar invasion. Invasion of strain *fig1*Δ *flo11*Δ is representative of all the other double-deletion mutants. This identifies Flo10 as the only other adhesin required for invasion in the absence of Flo11 in this analysis. Note that the granular nature of the macrocolonies is caused by flocculation in the cell suspensions after being dropped on the plate as S288C (*FLO8*) cells display strong flocculation.

**Figure 6:**
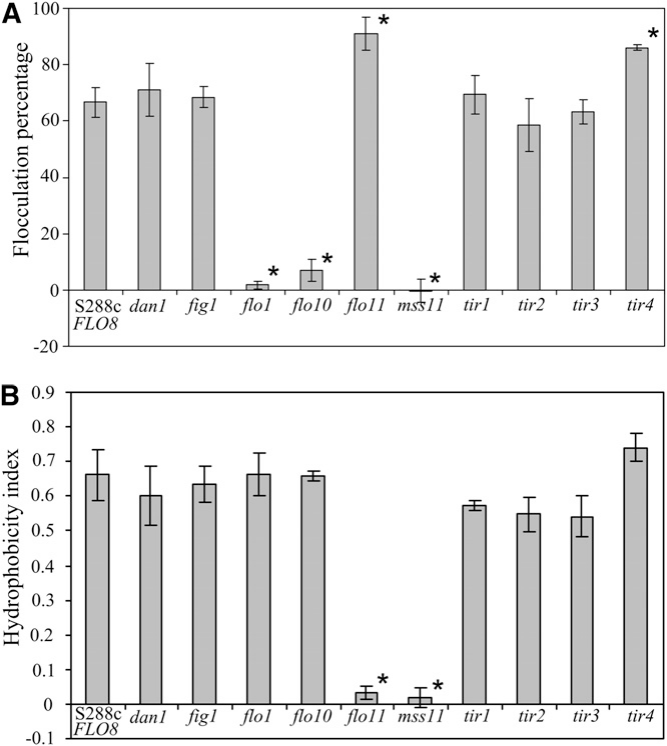
Degree of flocculation (A) and hydrophobicity (B) of S288c (*FLO8*) single-deletion mutants grown to stationary phase in liquid YPD. Mutant strains displaying a significant difference to wild type (*P* < 0.05) are indicated (*).

We further tested if single deletions of *DAN1*, *FIG1*, *FLO1*, *FLO10*, *FLO11*, *MSS11*, *TIR1*, *TIR2*, *TIR3*, and *TIR4* affect flocculation and cell hydrophobicity in S288c (*FLO8*) (Figure 6, A and B). Flocculation ability of strains grown to stationary phase was tested in YPD. Strains carrying deletions in *FLO1* and *MSS11* show no flocculation, thus confirming previous findings (Bester *et al.* 2006). We further identify the significant requirement of Flo10 as a flocculation factor as *FLO10* deletion leads to near total abolishment of flocculation. This requirement was not observed for *flo10*Δ strains grown in minimal media (data not shown); thus, this effect is very likely dependent on media composition. *FLO11* and *TIR4* deletions lead to a small but significant increase in flocculation. The cell wall hydrophobicity of the same strains is shown in Figure 6B. Deletion of *FLO11* or *MSS11* resulted in more hydrophilic cells supporting previous findings (Barrales *et al.* 2008) with no significant changes observed for the other deletion strains.

*FLO* proteins contain internal tandem repeats that display length variation between different strains and directly affect adhesin phenotypes (Verstrepen *et al.* 2005). Furthermore, yeast progeny from a common parental strain show great variation in flocculation (Smukalla *et al.* 2008) likely because of variation in the *FLO1* internal repeat region. To rule out the possibility that *FLO1* repeat variability is responsible for the discrepancies in flocculation observed in Figure 6, we amplified the repeat lengths from genomic DNA as described previously (Verstrepen *et al.* 2005) from the same strains used for the phenotype assay and found that all strains contained the same length repeat regions as compared with wild-type S288c (*FLO8*) (Figure S4).

### Differential regulation of *FLO10* and *FLO11*

**Figure 7:**
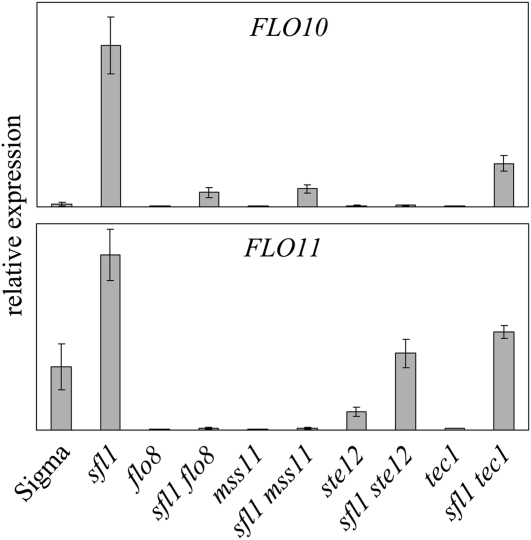
*FLO10* and *FLO11* are differentially regulated by components of the mitogen-activated protein kinase and cAMP-PKA signaling pathways: The relative expression of either *FLO10* or *FLO11* in wild-type strain Σ1278b (labeled Sigma) as well as single and double signaling mutants in the same genetic background.

We further investigated the possibility of *FLO1* and *FLO10* being regulated similarly. Σ1278b strains carrying single and double deletions in genes encoding for *FLO11* transcriptional control components were analyzed for *FLO* transcripts by means of qPCR analysis (Figure 7). Van Dyk *et al.* (2005) demonstrated that the absence of the Sfl1 repressor leads to the induction of *FLO11* transcription. This is blocked by a deletion of *FLO8*, acting down-stream of the cAMP-PKA pathway but only partially in yeast deleted for *STE12* or *TEC1*, which function downstream of the MAPK pathway. Our analysis confirms these findings for *FLO11* regulation and provides novel data for *FLO10* using cDNA from the same set of yeast strains (Figure 7). No significant levels of transcript of *FLO1* could be detected in wild-type or the deletion strains, supporting previous findings that this gene is silenced in Σ1278b (Guo *et al.* 2000). In the wild-type strain, *FLO10* transcript is lower compared with *FLO11* and the gene appears also repressed by Sfl1. It is partially dependent on the cAMP-PKA pathway, as can be seen from the signal still present in the *sfl1*Δ *flo8*Δ double mutant. No transcription could be detected in the *sfl1*Δ *ste12*Δ double mutant, suggesting that *FLO10* transcription also requires MAPK signaling. The transcript is still detected in the *sfl1*Δ *tec1*Δ strain, suggesting very specific roles of these MAPK pathway components in the regulation of *FLO10*. Transcription levels in *sfl1*Δ *mss11*Δ were the same as for the *sfl1*Δ *flo8*Δ strain.

## Discussion

### Mss11 affects multiple cell wall genes

In this study we show that the manipulation of *MSS11* expression levels has a significant impact on a number of genes encoding for cell wall related proteins. This assessment holds true for two strains that are geno- and phenotypically divergent, suggesting that Mss11 function is indeed more specifically related to cell wall remodeling. The data also clearly indicate that only *FLO1*, *FLO10*, and *FLO11* appear to be directly involved in the Mss11-controlled adhesion phenotypes that were assessed here, and that the other co-regulated genes have no specific role in such processes. This leaves the question of the specific roles of these proteins unanswered.

Interestingly in strain S288c, although significantly up-regulating many cell wall protein encoding genes, *MSS11* overexpression also had a significant repressive impact on the mating-related genes *FIG1* and *FIG2*. It is noteworthy that Fig1may also play a role in mating-unrelated polarized growth because it was shown that a *FIG1* transposon insertion mutant displayed decreased filamentation in response to 1-butanol (Lorenz *et al.* 2000). The data therefore suggest that Mss11 may play a role in directing cellular differentiation toward nonsexual adhesive phenotypes while repressing mating. It is possible that such a function is of some relevance in an evolutionary framework, similar to what has been observed in the case of *RME1* (Magwene *et al.* 2011; Van Dyk *et al.* 2003). In a nutrient-poor environment, mating may be undesirable even in the presence of mating partners because it certainly represents an energetically demanding and potentially risky exercise in such unfavorable conditions. Such an interpretation is reinforced by the fact that *MSS11* overexpression activates the high-affinity hexose transporter genes, *HXT2* (3.3 log_2_fc), *HXT4* (3.0 log_2_fc), and HXT6/7 (2.5 log_2_fc) in S288c (data not shown). Furthermore Mss11 controls starch use by the induction of glucoamylase-encoding *STA* genes that are found in some strains of *S. cerevisiae* (Gagiano *et al.* 1999a; Webber *et al.* 1997). Thus, Mss11 may be part of the switch between the mating and the adhesive, invasive or nutrient scavenging growth forms of *S. cerevisiae*.

Besides the *FLO* gene family, the *TIR* genes appear to be the second most *MSS11*-affected gene family. Indeed, several members of the group clearly and strongly respond to *MSS11* expression levels. However, our data do not add significantly to a better understanding of these genes. Indeed, none of the phenotypes investigated here were affected by deletions of these genes in both wild-type and *flo11*Δ genetic backgrounds. It is likely that other conditions will need to be investigated to find Mss11-dependent observable phenotypes associated with these genes.

### Adhesion phenotypes are dependent on multiple *FLO* genes

Our results show that the magnitude of specific phenotypes depends on more than one adhesin. In the strain S288c (*FLO8*), Flo1 is the dominant flocculation protein. However, Flo10 clearly plays a role in the process, and the absence of Flo11 leads to enhanced floc formation. Previously, it was reported that a *ssn6* strain with elevated expression levels of both *FLO1* (Fleming and Pennings 2001) and *FLO11* (Conlan and Tzamarias 2001) displays flocculent behavior, suggesting that Flo1 might be dominant over Flo11 (Conlan and Tzamarias 2001). High Mss11 levels similarly result in greater expression of *FLO1* and *FLO11*, but lead to both increased flocculation and invasion (Bester *et al.* 2006). It would be interesting to conduct controlled *FLO1* and *FLO11* co-expression and observe the phenotypic outcome, as that might shed some light on specific adhesin dominance and competitive or cooperative interactions between different adhesins. This study shows that agar invasion is dependent on both *FLO10* and *FLO11* and that Flo11 clearly is the dominant factor required for this behavior. Previous work has highlighted the level of functional overlap between Flo proteins by means of controlled or over-expression studies (Govender *et al.* 2008; Guo *et al.* 2000; Van Mulders *et al.* 2009). Results from this study strongly suggest that only *FLO* family members control cellular adhesion properties.

However, we did not investigate the effect of potential redundancy within gene families. For example, *TIR* gene family members display varying degrees of sequence homology. Different Tir proteins therefore are likely to show some functional overlap and may be able to complement phenotypes of single gene deletions. Thus, future work should focus on whole gene family deletions to rule out gene complementation. However, *FLO11* overexpression did not lead to any changes in *TIR* gene expression (Figure 3), yet resulted in the full range of adhesion-associated phenotypes associated with this protein. A specific role for Tir proteins in adhesion phenotypes therefore appears unlikely.

### Significance of non-*FLO* targets of *MSS11* overexpression

The specific roles of other cell wall encoding genes that are regulated by Mss11 remain unknown. Our data show that none of the tested genes directly interferes or impacts on *FLO11*-dependent phenotypes. Flo protein expression, on the other hand, does not appear to directly impact on the regulation of these genes, further suggesting that these genes have other, as yet-unknown roles that are unrelated to adhesion. Further phenotypic screening of mutant strains as well as strains carrying deletions of whole gene families may lead to some indication of such function.

Our data clearly show that overexpression of *FLO11* has little direct impact on other cell wall protein encoding genes. *FLO1*, *TIR1*, *TIR2*, *TIR3*, and *TIR4* are induced specifically in response to high Mss11 levels. In contrast to this *NCA3*, involved with cellular energy metabolism, is induced in response to both high Flo11 and Mss11 levels. This result suggests that high Flo11 levels may have metabolic impacts, either through sensing pathways responding to cell wall status or by indirectly changing the environment of individual cells from free-floating to being attached to substrates or other cells. However, considering that Σ1278b displays low levels of flocculation it is less likely that indirect gene activation in response to *MSS11* overexpression is the result of floc formation as reported by Smukalla *et al.* (2008).

We also show that high Flo11 levels clearly repress *AQY2* expression. This appears to be in contrast with findings by Furukawa *et al.* (2009), showing that Aqy2 affects adhesion phenotypes and cell wall characteristics in a similar fashion to Flo11p, and that *AQY2* regulation is similar to that of *FLO11*. Taken together, these findings suggest that there is a biological link between these two factors which results in differential regulation dependent on the specific experimental conditions.

### The regulation of *FLO10*

*FLO10*, similar to *FLO1* and *FLO11*, displays strong dependency on Mss11, although not to the same degree. Furthermore, *FLO10* responds to the same signaling pathways as *FLO11*. Our data show that *FLO10* transcription appears absolutely dependent on MAPK signaling since Ste12 is essential for its expression. Previous studies have shown that Ste12 and Tec1 regulate the *FLO11* promoter (Lo and Dranginis 1998; Madhani and Fink 1997) with Ste12 acting as the general MAPK signaling component and Tec1 as specific filamentous growth transcription factor (Bardwell *et al.* 1998; Madhani and Fink 1997). We show that these factors have different roles and requirements in the regulation of the *FLO10* promoter because the *sfl1*Δ *tec1*Δ mutant displays low levels of *FLO10* transcription. Thus *FLO10* transcriptional activation requires MAPK signaling but does not depend on the filamentous growth specific MAPK component Tec1.

It is surprising that the co-regulation of *FLO* genes with many other cell wall encoding genes appears to have no detectable impact on any of the relevant cell wall-dependent phenotypes investigated here. Indeed, no significant influence of any of these genes on the intensity of phenotypes such as flocculation (with the possible exception of TIR4), agar invasion or cell wall hydrophobicity was observed. These data strongly suggest that differential *FLO* gene regulation, controlled by overlapping pathways, is responsible for the balance of Flo proteins in the cell wall and that this balance is primarily responsible for governing the adhesion properties of the cell.

## Supplementary Material

Supporting Information
