# Supplementary material for: Many Saccharomyces cerevisiae Cell Wall Protein Encoding Genes Are Coregulated by Mss11, but Cellular Adhesion Phenotypes Appear Only Flo Protein Dependent
Source: G3 (Bethesda). 2012 Jan 1;2(1):131–41. doi: 10.1534/g3.111.001644 (PMC3276193; doi:10.1534/g3.111.001644)
Supplement: Supporting Information [file supp_2_1_131__index.html]

Supporting Information 

# Many *Saccharomyces cerevisiae* Cell Wall Protein Encoding Genes Are Coregulated by Mss11, but Cellular Adhesion Phenotypes Appear Only Flo Protein Dependent

## Supporting Information for Bester, Jacobson, and Bauer, 2012

**Files in this Data Supplement:**

- Supporting Information - Figures S1-S4 and Tables S1-S7 (PDF, 2.4 MB)
- Figure S1 - DAN1 transcript is only detected in strains over-expressing MSS11 as determined by qPCR (PDF, 232 KB)
- Figure S2 - Adhesion phenotype analysis of strains Σ1278b (labeled "Sigma"), S288c and S288c (FLO8) respectively (PDF, 1.4 MB)
- Figure S3 - Adhesion phenotypes of strains either over-expressing FLO11 or MSS11 (PDF, 244 KB)
- Figure S4 - FLO1 intragenic tandem repeat analysis showing repeats are of similar size in the single deletion strain set (PDF, 360 KB)
- Table S1 - Plasmids used in this study (PDF, 60 KB)
- Table S2 - S. cerevisiae strains used in this study (PDF, 84 KB)
- Table S3 - Primers used for the PCR amplification of gene disruption cassettes (PDF, 244 KB)
- Table S4 - Primers and hydrolysis probes used for qPCR analysis (PDF, 52 KB)
- Table S5 - Genes significantly regulated in response to MSS11 over-expression or deletion in ∑1278b (indicated as Σ1278b MSS11 and Σ1278b mss11 respectively) or over-expression in S288c (S288c MSS11) (PDF, 108 KB)
- Table S6 - GO enrichment analysis of genes significantly regulated in response to MSS11 over-expression or deletion in ∑1278b (indicated as Σ1278b MSS11 and Σ1278b mss11 respectively) or over-expression in S288c (S288c MSS11) (PDF, 86 KB)
- Table S7 - GO enrichment analysis of genes significantly regulated in response to FLO11 over-expression in ∑1278b (indicated as Σ1278b FLO11) using the online application FunSpec (p<0.0001; (Robinson et al. 2002) (PDF, 76 KB)
