# Supplementary material for: Many Saccharomyces cerevisiae Cell Wall Protein Encoding Genes Are Coregulated by Mss11, but Cellular Adhesion Phenotypes Appear Only Flo Protein Dependent
Source: G3 (Bethesda). 2012 Jan 1;2(1):131–41. doi: 10.1534/g3.111.001644 (PMC3276193; doi:10.1534/g3.111.001644)
Supplement: Supporting Information [file supp_2.1.131_001644SI.pdf]

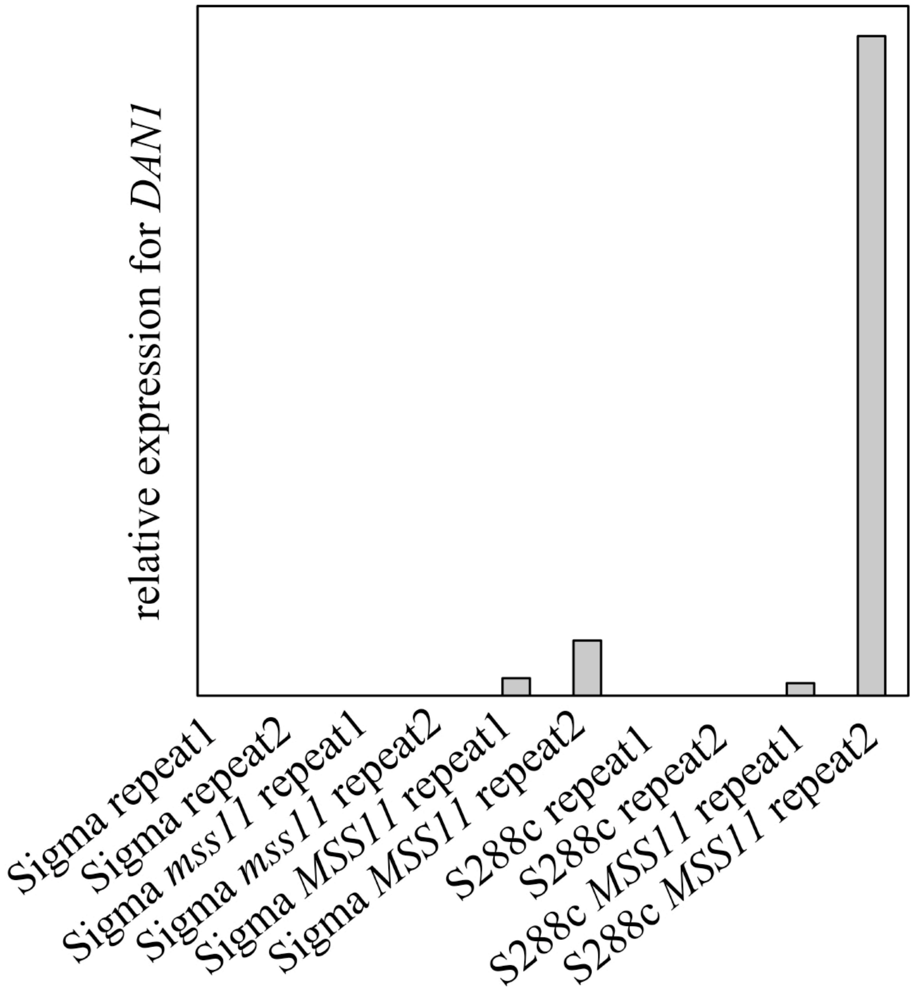

Figure S1 *DAN1* transcript is only detected in strains over-expressing *MSS11* as determined by qPCR. Shown is the relative expression data of each individual repeat for Σ1278b and S288c wild type strains (labeled Sigma and S288c respectively), Σ1278b with a *MSS11* deletion (Sigma *mss11*) and strains over-expressing *MSS11* (Sigma *MSS11* and S288c *MSS11*). Except for the over-expression strains no signal could be detected corresponding to *DAN1* transcript.

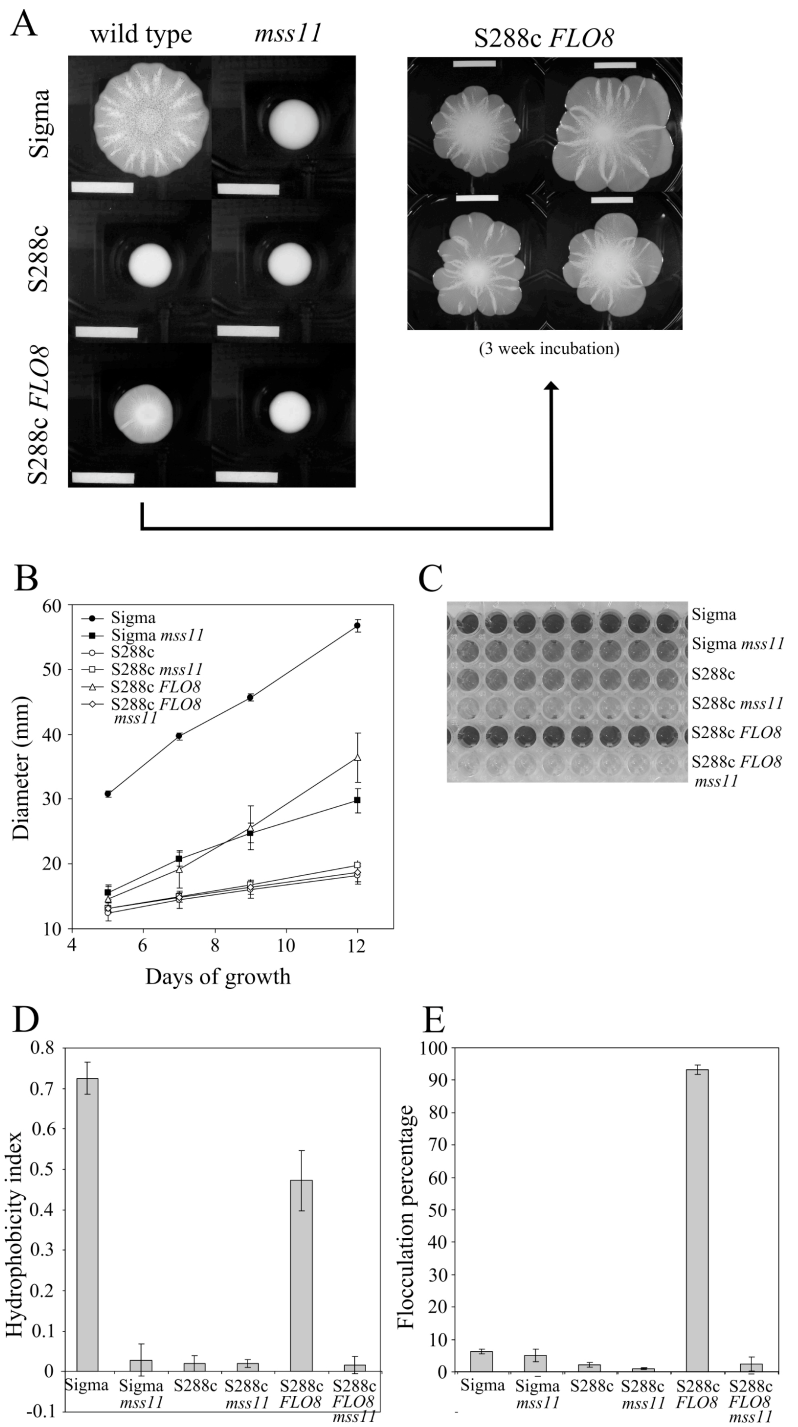

Figure S2 Adhesion phenotype analysis of strains Σ1278b (labeled "Sigma"), S288c and S288c (FLO8) respectively. Various cell-cell and cell-substrate interactions were investigated for wild type and MSS11 deletion (mss11) strains. (A) "Mat" formation on 0.3% YPD agar after 9 days of growth (left panel). Strain S288c (FLO8) was further incubated for ~2 weeks to allow for fully developed "mats" (right panel). Growth was measured up to 12 days post seeding as described in Materials and Methods (B). Strains grown to stationary phase in YPD were assayed for their ability to adhere to polystyrene (C), their degree of hydrophobicity (D) and for flocculation (E).

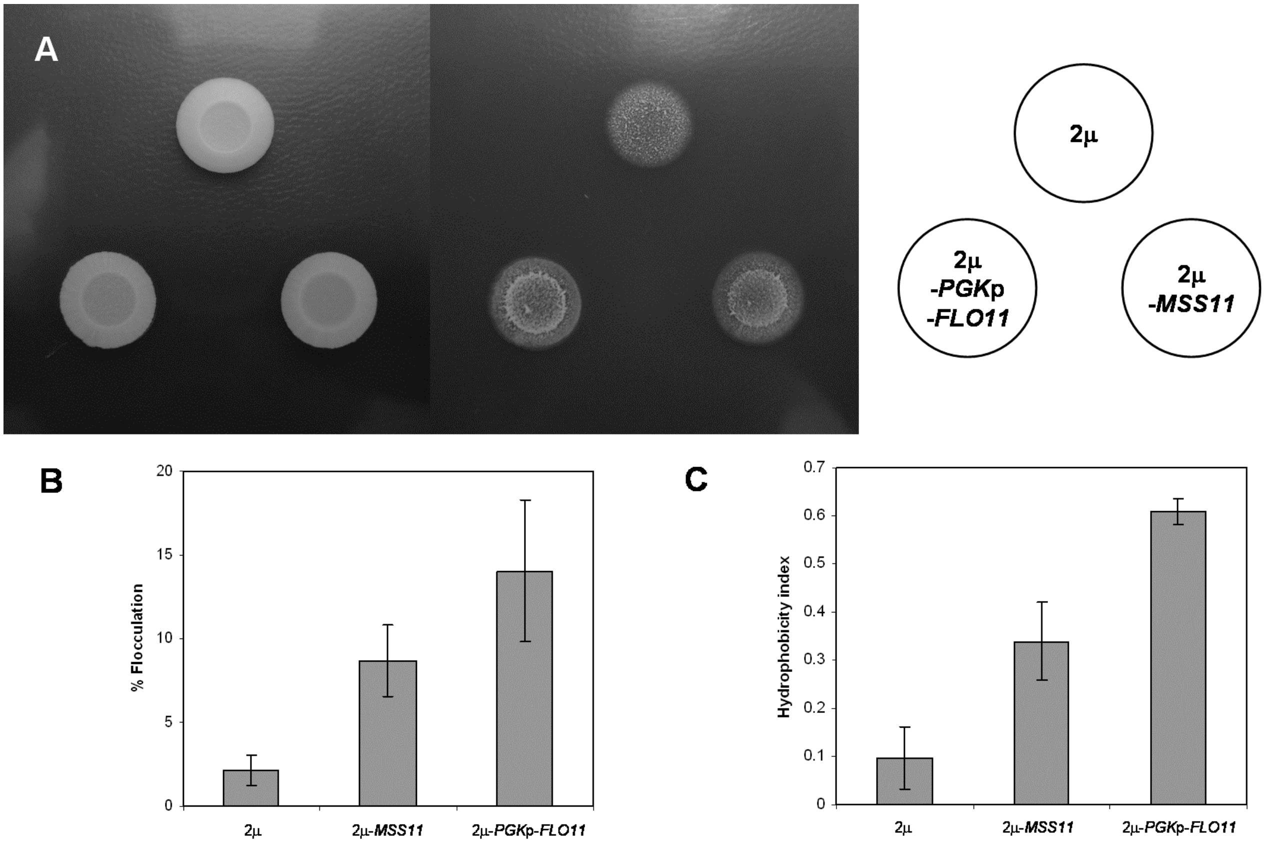

Figure S3 Adhesion phenotypes of strains either over-expressing *FLO11* or *MSS11*. Shown is (A) the ability to invade SCD agar plates following incubation of 12 days, (B) the degree of flocculent behavior and (C) outer cell hydrophobicity of transformants grown to stationary growth phase.

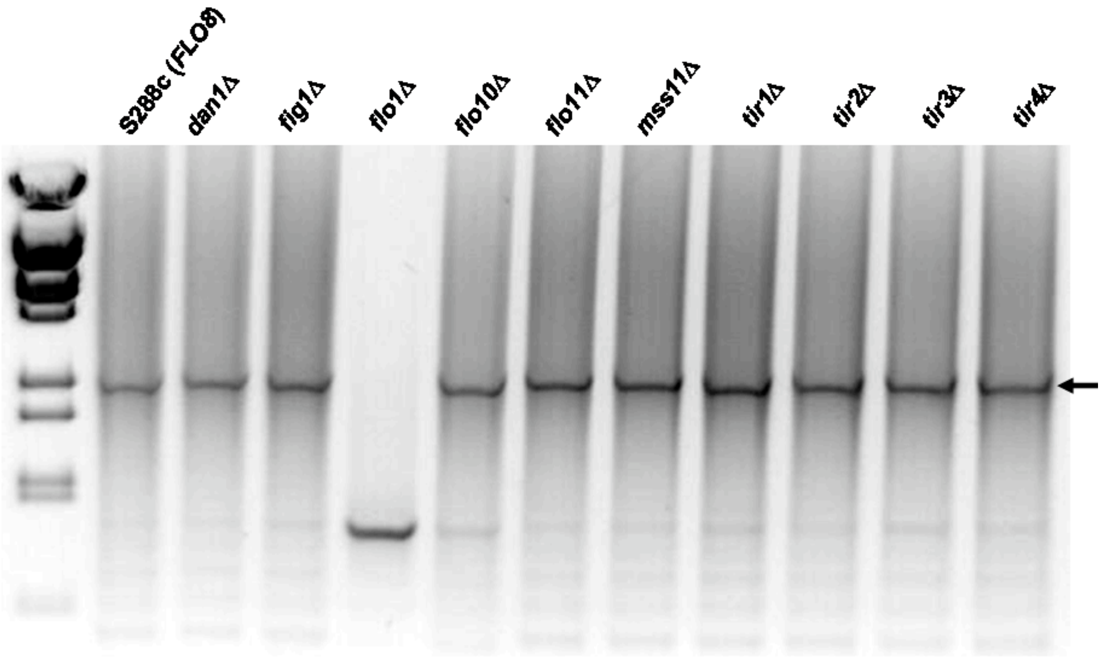

Figure S4 *FLO1* intragenic tandem repeat analysis showing repeats are of similar size in the single deletion strain set. *FLO1* repeats were PCR amplified using primers as described before (Verstrepen *et al.* 2005). DNA marker is lamda DNA digested with the restriction endo-nuclease *Bst*EII.

Table S1 Plasmids used in this study

Plasmid name	Relevant genotype	Source or reference
YEpLac181	2μ *LEU2*	(Gietz and Sugino 1988)
YEpLac195	2μ *URA3*	(Gietz and Sugino 1988)
YEpLac181-MSS11	2μ *LEU2 MSS11*	(Gagiano *et al.* 1999a)
YEpLac181-PGKp-MUC1	2μ *LEU2 PGK1*promoter *FLO11*	this lab
YEpLac195-MSS11	2μ *URA3 MSS11*	(Gagiano *et al.* 1999b)

Table 2 ***S. cerevisiae*** **strains used in this study**

Strain	Relevant genotype	Source or reference
BY4742	*MAT*α *his3*Δ1 *leu2*Δ0 *lys2*Δ0 *ura3*Δ0	(Brachmann *et al.* 1998)
BY4742 *dan1*Δ	isogenic to BY4742 *dan1*Δ::*KanMX4*	EUROSCARF
BY4742 *fig1*Δ	isogenic to BY4742 *fig1*Δ::*KanMX4*	EUROSCARF
BY4742 *fig2*Δ	isogenic to BY4742 *fig2*Δ::*KanMX4*	EUROSCARF
BY4742 *flo1*Δ	isogenic to BY4742 *flo1*Δ::*KanMX4*	EUROSCARF
BY4742 *flo10*Δ	isogenic to BY4742 *flo10*Δ::*KanMX4*	EUROSCARF
BY4742 *mss11*Δ	isogenic to BY4742 *mss11*Δ::*KanMX4*	EUROSCARF
BY4742 *nca3*Δ	isogenic to BY4742 *nca3*Δ::*KanMX4*	EUROSCARF
BY4742 *tir1*Δ	isogenic to BY4742 *tir1*Δ::*KanMX4*	EUROSCARF
BY4742 *tir2*Δ	isogenic to BY4742 *tir2*Δ::*KanMX4*	EUROSCARF
BY4742 *tir3*Δ	isogenic to BY4742 *tir3*Δ::*KanMX4*	EUROSCARF
BY4742 *tir4*Δ	isogenic to BY4742 *tir4*Δ::*KanMX4*	EUROSCARF
BY4742 *FLO8*	isogenic to BY4742 *flo8-1*Δ::*FLO8-LEU2*	(Bester *et al.* 2006)
BY4742 *FLO8 dan1*Δ	isogenic to BY4742 *dan1*Δ::*KanMX4 flo8-1*Δ::*FLO8-LEU2*	This study
BY4742 *FLO8 fig1*Δ	isogenic to BY4742 *fig1*Δ::*KanMX4 flo8-1*Δ::*FLO8-LEU2*	This study
BY4742 *FLO8 flo1*Δ	isogenic to BY4742 *flo8-1*Δ::*FLO8-LEU2 flo1*Δ::*KanMX4*	(Bester *et al.* 2006)
BY4742 *FLO8 flo10*Δ	isogenic to BY4742 *flo8-1*Δ::*FLO8-LEU2 flo10*Δ::*KanMX4*	(Bester *et al.* 2006)
BY4742 *FLO8 flo11*Δ	isogenic to BY4742 *flo8-1*Δ::*FLO8-LEU2 flo11*Δ::*lacZ-HIS3*	(Bester *et al.* 2006)
BY4742 *FLO8 tir1*Δ	isogenic to BY4742 *tir1*Δ::*KanMX4 flo8-1*Δ::*FLO8-LEU2*	This study
BY4742 *FLO8 tir2*Δ	isogenic to BY4742 *tir2*Δ::*KanMX4 flo8-1*Δ::*FLO8-LEU2*	This study
BY4742 *FLO8 tir3*Δ	isogenic to BY4742 *tir3*Δ::*KanMX4 flo8-1*Δ::*FLO8-LEU2*	This study

BY4742 *FLO8 tir4Δ*	isogenic to BY4742 *tir4Δ::KanMX4 flo8-1Δ::FLO8-LEU2*	This study
BY4742 *FLO8 dan1Δ flo11Δ::lacZ*	isogenic to BY4742 *dan1Δ::KanMX4 flo8-1Δ::FLO8-LEU2 flo11Δ::lacZ-HIS3*	This study
BY4742 *FLO8 fig1Δ flo11Δ::lacZ*	isogenic to BY4742 *fig1Δ::KanMX4 flo8-1Δ::FLO8-LEU2 flo11Δ::lacZ-HIS3*	This study
BY4742 *FLO8 fig2Δ flo11Δ::lacZ*	isogenic to BY4742 *fig2Δ::KanMX4 flo8-1Δ::FLO8-LEU2 flo11Δ::lacZ-HIS3*	This study
BY4742 *FLO8 flo1Δ flo11Δ::lacZ*	isogenic to BY4742 *flo1Δ::KanMX4 flo8-1Δ::FLO8-LEU2 flo11Δ::lacZ-HIS3*	This study
BY4742 *FLO8 flo10Δ flo11Δ::lacZ*	isogenic to BY4742 *flo10Δ::KanMX4 flo8-1Δ::FLO8-LEU2 flo11Δ::lacZ-HIS3*	This study
BY4742 *FLO8 flo11Δ::lacZ nca3Δ*	isogenic to BY4742 *nca3Δ::KanMX4 flo8-1Δ::FLO8-LEU2 flo11Δ::lacZ-HIS3*	This study
BY4742 *FLO8 flo11Δ::lacZ tir1Δ*	isogenic to BY4742 *tir1Δ::KanMX4 flo8-1Δ::FLO8-LEU2 flo11Δ::lacZ-HIS3*	This study
BY4742 *FLO8 flo11Δ::lacZ tir2Δ*	isogenic to BY4742 *tir2Δ::KanMX4 flo8-1Δ::FLO8-LEU2 flo11Δ::lacZ-HIS3*	This study
BY4742 *FLO8 flo11Δ::lacZ tir3Δ*	isogenic to BY4742 *tir3Δ::KanMX4 flo8-1Δ::FLO8-LEU2 flo11Δ::lacZ-HIS3*	This study
BY4742 *FLO8 flo11Δ::lacZ tir4Δ*	isogenic to BY4742 *tir4Δ::KanMX4 flo8-1Δ::FLO8-LEU2 flo11Δ::lacZ-HIS3*	This study
YHUM272	*MATα ura3-52 trp1Δ::hisG leu2Δ::hisG his3Δ::hisG*	H.-U. Mösch
Σ1278b *flo8*	isogenic to Σ1278b *flo8Δ::LEU2*	(van Dyk et al. 2005)
Σ1278b *flo11*	isogenic to Σ1278b *flo11Δ::lacZ-HIS3*	(van Dyk et al. 2005)
Σ1278b *mss11*	isogenic to Σ1278b *mss11Δ::LEU2*	(van Dyk et al. 2005)
Σ1278b *sfl1*	isogenic to Σ1278b *sfl1Δ::KanMX4*	(van Dyk et al. 2005)
Σ1278b *ste12*	isogenic to Σ1278b *ste12Δ::URA3*	(van Dyk et al. 2005)
Σ1278b *tec1*	isogenic to Σ1278b *tec1Δ::LEU2*	(van Dyk et al. 2005)
Σ1278b *sfl1 flo8*	isogenic to Σ1278b *sfl1Δ::KanMX4 flo8Δ::LEU2*	(van Dyk et al. 2005)
Σ1278b *sfl1 mss11*	isogenic to Σ1278b *sfl1Δ::KanMX4 mss11Δ::LEU2*	(van Dyk et al. 2005)
Σ1278b *sfl1 ste12*	isogenic to Σ1278b *sfl1Δ::KanMX4 ste12Δ::URA3*	(van Dyk et al. 2005)
Σ1278b *sfl1 tec1*	isogenic to Σ1278b *sfl1Δ::KanMX4 tec1Δ::LEU2*	(van Dyk et al. 2005)
Σ1278b *dan1Δ flo11Δ::lacZ*	isogenic to Σ1278b *dan1Δ::KanMX4 flo11Δ::lacZ-HIS3*	This study
Σ1278b *fig1Δ flo11Δ::lacZ*	isogenic to Σ1278b *figΔ::KanMX4 flo11Δ::lacZ-HIS3*	This study

Σ1278b *fig2*Δ *flo11*Δ::*lacZ*	isogenic to Σ1278b *fig2*Δ::*KanMX4 flo11*Δ::*lacZ-HIS3*	This study
Σ1278b *flo1*Δ *flo11*Δ::*lacZ*	isogenic to Σ1278b *flo1*Δ::*KanMX4 flo11*Δ::*lacZ-HIS3*	This study
Σ1278b *flo10*Δ *flo11*Δ::*lacZ*	isogenic to Σ1278b *flo10*Δ::*KanMX4 flo11*Δ::*lacZ-HIS3*	This study
Σ1278b *flo11*Δ::*lacZ nca3*Δ	isogenic to Σ1278b *nca3*Δ::*KanMX4 flo11*Δ::*lacZ-HIS3*	This study
Σ1278b *flo11*Δ::*lacZ tir1*Δ	isogenic to Σ1278b *tir1*Δ::*KanMX4 flo11*Δ::*lacZ-HIS3*	This study
Σ1278b *flo11*Δ::*lacZ tir2*Δ	isogenic to Σ1278b *tir2*Δ::*KanMX4 flo11*Δ::*lacZ-HIS3*	This study
Σ1278b *flo11*Δ::*lacZ tir3*Δ	isogenic to Σ1278b *tir3*Δ::*KanMX4 flo11*Δ::*lacZ-HIS3*	This study
Σ1278b *flo11*Δ::*lacZ tir4*Δ	isogenic to Σ1278b *tir4*Δ::*KanMX4 flo11*Δ::*lacZ-HIS3*	This study

Table S3 Primers used for the PCR amplification of gene disruption cassettes

Primer name	Primer sequence (5'→3')
AQY2kanMX4-F	CTTTCATTAACGAATTAGAGCGC
AQY2kanMX4-R	TCAAAGCCATGTGAGCCATG
DAN1kanMX4-F	CTCCGTAGACGCTCCTCTGAA
DAN1kanMX4-R	TGTTAAGCTGTCTGCAATAAGGAAT
FIG1kanMX4-F	GATGGTTTCATGTATGTGTCAGTTAAA
FIG1kanMX4-R	GTCGCTCATCAAGGTGACAGTAA
FIG2kanMX4-F	GGTACATGGTGCTTTCCTTATGC
FIG2kanMX4-R	GCCTTGATTGCAGAGGTTGTG
FLO1-Fp-Conf	CGATAGGGAGGCATCATGGTACTACCG
FLO1-Rp-Conf	AAGAAGCGCAAGAATTATCATTTAGTCAAT
FLO10-F	AAATGGGCTCCTGCCTGAAT
FLO10-R	CTAGCTCATCCGTTGCCGCT
HPF1(YOL155c)kanMX4-F	GCAGAAGTGCCCGTAGGAGA
HPF1(YOL155c)kanMX4-R	GTGGACAATAAGTGAAATAAGTGTCCTT
NCA3kanMX4-F	GTGGCAAAGCGGACAGCT
NCA3kanMX4-R	CCGTAATGCAGAGTACACCTTGA
TIR1kanMX4-F	GAACTGCGTTTGTATGCAACTGT
TIR1kanMX4-R	GTATCCAACAGACAGTAGTGCCAAC
tir2kof	GCTGAGCATCATGTGAGTAA
tir2kor	ATGTGGGCAGGAAGACATGC
tir3kof	GCCCTAGAATGGATTGCAGA
tir3kor	TGCTCTTGCTCGAACATTCC
TIR4kanMX4-F	GCATTTCTAACAAGTAGGATAGTCCAA
TIR4kanMX4-R	GATGATGCTAGGATAGGCACCTT

Table S4 Primers and hydrolysis probes used for qPCR analysis

Primer/probe name	Primer/probe sequence (5’→3’)	Modifications
FLO9-F (TaqmanMGB)	TGTACAATAAAAGCCCCAAAAATG	none
FLO9-R (TaqmanMGB)	GCAATGTGACGATGGCTAGTAGTAA	none
FLO9-probe	CTCTGGCACATTATT	NED dye (5'), Minor Groove Binder (3')
AQY2-F-(rt-PCR)	GGACCCGACCGGTGTTG	none
AQY2-R-(rt-PCR)	TTAAAACGCGAATGCTTCGTT	none
DAN1-F-(rt-PCR)	GCTTTCCAGGCTTTGCATAAGA	none
DAN1-R-(rt-PCR)	TCGCCACCGGCAAAAA	none
DAN4-F-(rt-PCR)	GCCACTACATCGAACAATGCA	none
DAN4-R-(rt-PCR)	GGCACCCGCAGAGCAA	none
FIG1-F-(rt-PCR)	TCCCTTATACAGAGACTTGGAAATTCA	none
FIG1-R-(rt-PCR)	AATTGGGCTAACTTCAAAATGTTCA	none
FIG2-F-(rt-PCR)	CTTCTGATACTTTTTCTTCATACTCTGATATCT	none
FIG2-R-(rt-PCR)	TGTCCTATGAGGTTGTGCAGTTG	none
FLO11-F-(QRT-PCR)	CCTCCGAAGGAACTAGCTGTAATT	none
FLO11-R-(QRT-PCR)	AGTCACATCCAAAGTATACTGCATGAT	none
HPF1(YIL169c)-F-(rt-PCR)	CTAAGGACATACACTACTGCCACTGGT	none
HPF1(YIL169c)-R-(rt-PCR)	ACTAGTTGCGTGACGGTTGAAGTAG	none
HPF1(YOL155c)-F-(rt-PCR)	CGGTTCATCTTCTGCCACAGA	none
HPF1(YOL155c)-R-(rt-PCR)	GTTCATCTTCTGCCACAGAATCAG	none
NCA3-F-(rt-PCR)	TGGTGGATGGGCCTCTGT	none
NCA3-R-(rt-PCR)	GACATTCCAGGTTCACATGCA	none
PDA1-F-QRT-PCR	GGAATTTGCCCGTCGTGTT	none
PDA1-R-QRT-PCR	GCGGCGGTACCCATACC	none
TIR1-F-(rt-PCR)	TCCAAGCTACCAAGGCTGTTTC	none
TIR1-R-(rt-PCR)	ACCCATACCAACAAAGGCCTTA	none
TIR2-F-qPCR	CTGCCCAAACTCAGGAAGAAA	none
TIR2-R-qPCR	CTTGCAAGTTGGACTTAACGTCAT	none
TIR3-F-(QRT-PCR)	TTTGACGCTATTTTGGCTGATG	none

TIR3-R-(QRT-PCR)	TCTGGATTATTCATTGCCAAGGA	none
TIR4-F-(rt-PCR)	TGCCGACTACATCACCCTATCC	none
TIR4-R-(rt-PCR)	GGCATTTGGTCCAAGGAAAA	none

Table S5 Genes significantly regulated in response to *MSS11* over-expression or deletion in ∑1278b (indicated as Σ1278b *MSS11* and Σ1278b *mss11* respectively) or over-expression in S288c (S288c *MSS11*). Shown are the degree of regulation (log_2fold), the open reading frame (ORF), the gene name if applicable as well as a short description as obtained from the *Saccharomyces genome database* (SGD).

log_2fold	ORF	Gene name	Short description
S288c *MSS11*			
-2.738	YJL170C	*ASG7*	Protein that regulates signaling from a G protein beta subunit Ste4p and its relocalization within the cell
-2.307	YIL015W	*BAR1*	Aspartyl protease secreted into the periplasmic space of mating type a cells, helps cells find mating partners, cleaves and inactivates alpha factor allowing cells to recover from alpha-factor-induced cell cycle arrest
-1.87	YCL026C-A	*FRM2*	Protein of unknown function, involved in the integration of lipid signaling pathways with cellular homeostasis
-1.148	YMR279C		Putative protein of unknown function
-1.066	YJL160C		Putative protein of unknown function
-1.061	YDL127W	*PCL2*	G1 cyclin, associates with Pho85p cyclin-dependent kinase (Cdk) to contribute to entry into the mitotic cell cycle, essential for cell morphogenesis
-0.919	YBR019C	*GAL10*	UDP-glucose-4-epimerase, catalyzes the interconversion of UDP-galactose and UDP-D-glucose in galactose metabolism
-0.919	YOR114W		Putative protein of unknown function
-0.887	YPL130W	*SPO19*	Meiosis-specific protein of unknown function, involved in completion of nuclear divisions
-0.85	R0020C	*REP1*	Master regulator that acts in concert with Rep2p to regulate transcript levels of the FLP1 gene that promotes plasmid copy amplification
-0.849	YGR131W		Protein of unknown function
-0.807	YDR106W	*ARP10*	Component of the dynactin complex, localized to the pointed end of the Arp1p filament
-0.7	YJR112W	*NNF1*	Essential component of the MIND kinetochore complex (Mtw1p Including Nnf1p-Nsl1p-Dsn1p) which joins kinetochore subunits contacting DNA to those contacting microtubules
-0.655	YPR106W	*ISR1*	Predicted protein kinase, overexpression causes sensitivity to staurosporine, which is a potent inhibitor of protein kinase C

-0.651	YKL089W	*MIF2*	Kinetochore protein with homology to human CENP-C, required for structural integrity of the spindle during anaphase spindle elongation, interacts with histones H2A, H2B, and H4, phosphorylated by Ipl1p
-0.647	YJL196C	*ELO1*	Elongase I, medium-chain acyl elongase, catalyzes carboxy-terminal elongation of unsaturated C12-C16 fatty acyl-CoAs to C16-C18 fatty acids
-0.627	YDR179W-A		Putative protein of unknown function
-0.626	YHR063C	*PAN5*	2-dehydropantoate 2-reductase, part of the pantothenic acid pathway, structurally homologous to E. coli panE
-0.569	YFL046W	*FMP32*	Putative protein of unknown function
-0.513	YML023C	*NSE5*	Essential subunit of the Mms21-Smc5-Smc6 complex
0.5	YNR018W		Putative protein of unknown function
0.746	YLR216C	*CPR6*	Peptidyl-prolyl cis-trans isomerase (cyclophilin), catalyzes the cis-trans isomerization of peptide bonds N-terminal to proline residues
0.783	YHR112C		Putative protein of unknown function
0.859	YPL024W	*RMI1*	Subunit of the RecQ (Sgs1p) - Topo III (Top3p) complex
0.904	YNL191W	*DUG3*	Probable glutamine amidotransferase, forms a complex with Dug1p and Dug2p to degrade glutathione (GSH) and other peptides containing a gamma-glu-X bond in an alternative pathway to GSH degradation by gamma-glutamyl transpeptidase (Ecm38p)
0.966	YER042W	*MXR1*	Methionine-S-sulfoxide reductase, involved in the response to oxidative stress
1.039	YGL010W		Putative protein of unknown function
1.162	YPR196W		Putative maltose activator
1.225	YAL012W	*CYS3*	Cystathionine gamma-lyase, catalyzes one of the two reactions involved in the transsulfuration pathway that yields cysteine from homocysteine with the intermediary formation of cystathionine
1.249	YNL276C		
1.249	YNL277W	*MET2*	L-homoserine-O-acetyltransferase, catalyzes the conversion of homoserine to O-acetyl homoserine which is the first step of the methionine biosynthetic pathway

1.465	YDL059C	*RAD59*	Protein involved in the repair of double-strand breaks in DNA during vegetative growth via recombination and single-strand annealing
1.501	YDR044W	*HEM13*	Coproporphyrinogen III oxidase, an oxygen requiring enzyme that catalyzes the sixth step in the heme biosynthetic pathway
1.521	YHL036W	*MUP3*	Low affinity methionine permease, similar to Mup1p
1.528	YKL109W	*HAP4*	Subunit of the heme-activated, glucose-repressed Hap2p/3p/4p/5p CCAAT-binding complex, a transcriptional activator and global regulator of respiratory gene expression
1.599	YDL245C	*HXT15*	Protein of unknown function with similarity to hexose transporter family members, expression is induced by low levels of glucose and repressed by high levels of glucose
1.599	YJR158W	*HXT16*	Protein of unknown function with similarity to hexose transporter family members, expression is repressed by high levels of glucose
1.655	YOR009W	*TIR4*	Cell wall mannoprotein of the Srp1p/Tip1p family of serine-alanine-rich proteins
1.658	YLL061W	*MMP1*	High-affinity S-methylmethionine permease, required for utilization of S-methylmethionine as a sulfur source
1.719	YKL201C	*MNN4*	Putative positive regulator of mannosylphosphate transferase (Mnn6p), involved in mannosylphosphorylation of N-linked oligosaccharides
1.719	YKL202W		
1.755	YMR244W		Putative protein of unknown function
1.808	YLR364W	*GRX8*	Glutaredoxin that employs a dithiol mechanism of catalysis
1.881	YDR253C	*MET32*	Zinc-finger DNA-binding protein, involved in transcriptional regulation of the methionine biosynthetic genes, similar to Met31p
1.898	YMR081C	*ISF1*	Serine-rich, hydrophilic protein with similarity to Mbr1p
1.931	YJL212C	*OPT1*	Proton-coupled oligopeptide transporter of the plasma membrane
1.961	YFL051C		Putative protein of unknown function
1.968	YEL070W	*DSF1*	Deletion suppressor of mpt5 mutation
1.968	YNR073C		Putative mannitol dehydrogenase
2.1	YGR055W	*MUP1*	High affinity methionine permease, integral membrane protein with 13 putative membrane-spanning regions

2.374	YAR064W		Putative protein of unknown function
2.374	YHR213W-B		Putative protein of unknown function
2.458	YER011W	*TIR1*	Cell wall mannoprotein of the Srp1p/Tip1p family of serine-alanine-rich proteins
2.47	YDR342C	*HXT7*	High-affinity glucose transporter of the major facilitator superfamily, nearly identical to Hxt6p, expressed at high basal levels relative to other HXTs, expression repressed by high glucose levels
2.47	YDR343C	*HXT6*	High-affinity glucose transporter of the major facilitator superfamily, nearly identical to Hxt7p, expressed at high basal levels relative to other HXTs, repression of expression by high glucose requires SNF3
2.522	YKL068W-A		Putative protein of unknown function
2.629	YJL218W		Putative protein of unknown function, similar to bacterial galactoside O-acetyltransferases
2.951	YAL063C	*FLO9*	Lectin-like protein with similarity to Flo1p, thought to be expressed and involved in flocculation
2.951	YAL064W-B		Fungal-specific protein of unknown function
2.951	YAR050W	*FLO1*	Lectin-like protein involved in flocculation, cell wall protein that binds to mannose chains on the surface of other cells, confers floc-forming ability that is chymotrypsin sensitive and heat resistant
2.951	YHR211W	*FLO5*	Lectin-like cell wall protein (flocculin) involved in flocculation, binds to mannose chains on the surface of other cells, confers floc-forming ability that is chymotrypsin resistant but heat labile
2.966	YHR092C	*HXT4*	High-affinity glucose transporter of the major facilitator superfamily, expression is induced by low levels of glucose and repressed by high levels of glucose
3.052	YMR317W		Putative protein of unknown function with some similarity to sialidase from Trypanosoma
3.172	YMR164C	*MSS11*	Transcription factor involved in regulation of invasive growth and starch degradation
3.214	YDL039C	*PRM7*	Pheromone-regulated protein, predicted to have one transmembrane segment
3.23	YIL011W	*TIR3*	Cell wall mannoprotein of the Srp1p/Tip1p family of serine-alanine-rich proteins
3.319	YMR011W	*HXT2*	High-affinity glucose transporter of the major facilitator superfamily, expression is induced by low levels of glucose and repressed by

			high levels of glucose
3.732	YDL039C	*PRM7*	Pheromone-regulated protein, predicted to have one transmembrane segment
3.747	YAL065C		Putative protein of unknown function
3.822	YOR010C	*TIR2*	Putative cell wall mannoprotein of the Srp1p/Tip1p family of serine-alanine-rich proteins
3.853	YJR150C	*DAN1*	Cell wall mannoprotein with similarity to Tir1p, Tir2p, Tir3p, and Tir4p
3.876	YHR213W-A		Putative protein of unknown function
4.818	YJL116C	*NCA3*	Protein that functions with Nca2p to regulate mitochondrial expression of subunits 6 (Atp6p) and 8 (Atp8p) of the Fo-F1 ATP synthase
4.991	YIR019C	*MUC1*	GPI-anchored cell surface glycoprotein (flocculin) required for pseudohyphal formation, invasive growth, flocculation, and biofilms
5.373	YAR062W		Hypothetical protein
5.373	YHR213W		Putative protein of unknown function
5.798	YAR050W	*FLO1*	Lectin-like protein involved in flocculation, cell wall protein that binds to mannose chains on the surface of other cells, confers floc-forming ability that is chymotrypsin sensitive and heat resistant
Σ1278b *MSS11*			
-0.857	YOL147C	*PEX11*	Peroxisomal membrane protein required for peroxisome proliferation and medium-chain fatty acid oxidation, most abundant protein in the peroxisomal membrane, regulated by Adr1p and Pip2p-Oaf1p, promoter contains ORE and UAS1-like elements
-0.855	YHR143W	*DSE2*	Daughter cell-specific secreted protein with similarity to glucanases, degrades cell wall from the daughter side causing daughter to separate from mother
0.916	YNL036W	*NCE103*	Carbonic anhydrase
1.086	YGR213C	*RTA1*	Protein involved in 7-aminocholesterol resistance
1.204	YOR032C	*HMS1*	Basic helix-loop-helix (bHLH) protein with similarity to myc-family transcription factors
1.321	YMR317W		Putative protein of unknown function with some similarity to sialidase from Trypanosoma
1.321	YIL013C	*PDR11*	ATP-binding cassette (ABC) transporter, multidrug transporter involved in multiple drug resistance

1.403	YHR213W-A		Putative protein of unknown function
1.437	YAL063C	*FLO9*	Lectin-like protein with similarity to Flo1p, thought to be expressed and involved in flocculation
1.437	YAL064W-B		Fungal-specific protein of unknown function
1.437	YAR050W	*FLO1*	Lectin-like protein involved in flocculation, cell wall protein that binds to mannose chains on the surface of other cells, confers floc-forming ability that is chymotrypsin sensitive and heat resistant
1.437	YHR211W	*FLO5*	Lectin-like cell wall protein (flocculin) involved in flocculation, binds to mannose chains on the surface of other cells, confers floc-forming ability that is chymotrypsin resistant but heat labile
1.494	YDL039C	*PRM7*	Pheromone-regulated protein, predicted to have one transmembrane segment
1.528	YNR002C	*ATO2*	Putative transmembrane protein involved in export of ammonia, a starvation signal that promotes cell death in aging colonies
1.571	YHL043W	*ECM34*	Putative protein of unknown function
1.66	YOR009W	*TIR4*	Cell wall mannoprotein of the Srp1p/Tip1p family of serine-alanine-rich proteins
1.892	YMR081C	*ISF1*	Serine-rich, hydrophilic protein with similarity to Mbr1p
2.206	YAR050W	*FLO1*	Lectin-like protein involved in flocculation, cell wall protein that binds to mannose chains on the surface of other cells, confers floc-forming ability that is chymotrypsin sensitive and heat resistant
2.229	YJL116C	*NCA3*	Protein that functions with Nca2p to regulate mitochondrial expression of subunits 6 (Atp6p) and 8 (Atp8p) of the Fo-F1 ATP synthase
2.452	YAR062W		Hypothetical protein
2.452	YHR213W		Putative protein of unknown function
2.49	YIR019C	*MUC1*	GPI-anchored cell surface glycoprotein (flocculin) required for pseudohyphal formation, invasive growth, flocculation, and biofilms
2.586	YIL011W	*TIR3*	Cell wall mannoprotein of the Srp1p/Tip1p family of serine-alanine-rich proteins
2.791	YDL039C	*PRM7*	Pheromone-regulated protein, predicted to have one transmembrane segment
4.329	YMR164C	*MSS11*	Transcription factor involved in regulation of invasive growth and starch degradation
4.497	YOR010C	*TIR2*	Putative cell wall mannoprotein of the Srp1p/Tip1p family of serine-alanine-rich proteins

Σ1278b *mss11*			
-3.171	YIR019C	*MUC1*	GPI-anchored cell surface glycoprotein (flocculin) required for pseudohyphal formation, invasive growth, flocculation, and biofilms
-2.962	YMR164C	*MSS11*	Transcription factor involved in regulation of invasive growth and starch degradation
-2.426	YOR032C	*HMS1*	Basic helix-loop-helix (bHLH) protein with similarity to myc-family transcription factors
-2.199	YDR259C	*YAP6*	Putative basic leucine zipper (bZIP) transcription factor
-1.683	YIL011W	*TIR3*	Cell wall mannoprotein of the Srp1p/Tip1p family of serine-alanine-rich proteins
-1.656	YHL043W	*ECM34*	Putative protein of unknown function
-1.21	YPR013C		Putative zinc finger protein

Table S6 GO enrichment analysis of genes significantly regulated in response to *MSS11* over-expression or deletion in ∑1278b (indicated as Σ1278b *MSS11* and Σ1278b *mss11* respectively) or over-expression in S288c (S288c *MSS11*). Shown is the number of genes identified to group in each category as selected by the use of GOEast with a Benjamini & Yekutieli calculated FDR threshold of 0.1. Categories mentioned in text are shown in bold.

GO Term	GO ID	S288c *MSS11*	Sigma *MSS11*	Sigma *mss11*
DNA binding	GO:0003677			3
L-amino acid transmembrane transporter activity	GO:0015179	2		
amine transmembrane transporter activity	GO:0005275	3		
amino acid transmembrane transporter activity	GO:0015171	3		
amino acid transport	GO:0006865	3		
**anchored to membrane**	**GO:0031225**	**9**	**7**	**2**
carbohydrate binding	GO:0030246	3	2	
carbohydrate transmembrane transporter activity	GO:0015144	4		
carbohydrate transport	GO:0008643	4		
cell	GO:0005623			7
cell growth	GO:0016049		4	3
cell part	GO:0044464			7
**cell periphery**	**GO:0071944**	**16**	**8**	
**cell wall**	**GO:0005618**	**10**	**7**	**2**
cell wall organization	GO:0071555		3	
cellular cell wall organization	GO:0007047		3	
**external encapsulating structure**	**GO:0030312**	**10**	**7**	**2**
external encapsulating structure organization	GO:0045229		3	
**extracellular region**	**GO:0005576**	**11**	**7**	**2**
**filamentous growth**	**GO:0030447**		**4**	**3**
filamentous growth of a population of unicellular organisms	GO:0044182		4	3
**flocculation**	**GO:0000128**	**4**	**4**	**1**
flocculation via cell wall protein-carbohydrate interaction	GO:0000501	2	2	
fructose transmembrane transporter activity	GO:0005353	4		
**fungal-type cell wall**	**GO:0009277**	**7**	**5**	**1**
glucose transmembrane transporter activity	GO:0005355	4		

GO Term	GO ID	S288c *MSS11*	Sigma *MSS11*	Sigma *mss11*
growth	GO:0040007		4	3
growth of unicellular organism as a thread of attached cells	GO:0070783		4	3
hexose transmembrane transporter activity	GO:0015149	4		
hexose transport	GO:0008645	4		
homoserine metabolic process	GO:0009092	2		
**intrinsic to membrane**	**GO:0031224**	**30**	**13**	
mannose transmembrane transporter activity	GO:0015578	4		
membrane	GO:0016020	30	13	
membrane part	GO:0044425	30	13	
modified amino acid transmembrane transporter activity	GO:0072349	3		
monosaccharide binding	GO:0048029	2	2	
monosaccharide transmembrane transporter activity	GO:0015145	4		
monosaccharide transport	GO:0015749	4		
**multi-organism process**	**GO:0051704**	**7**	**5**	**1**
organic substance transport	GO:0071702	8		
peptide catabolic process	GO:0043171	2		
peptide metabolic process	GO:0006518	2		
**plasma membrane**	**GO:0005886**	**9**	**4**	
pseudohyphal growth	GO:0007124		4	3
regulation of anatomical structure size	GO:0090066		4	3
regulation of biological quality	GO:0065008			3
regulation of cell size	GO:0008361		4	3
regulation of cellular component size	GO:0032535		4	3
response to stimulus	GO:0050896		10	
response to toxin	GO:0009636	2	1	
sequence-specific DNA binding	GO:0043565			2
sugar binding	GO:0005529	2	2	
sugar transmembrane transporter activity	GO:0051119	4		
sulfur amino acid metabolic process	GO:0000096	3		
sulfur amino acid transmembrane transporter activity	GO:0000099	3		

GO Term	GO ID	S288c *MSS11*	Sigma *MSS11*	Sigma *mss11*
sulfur compound metabolic process	GO:0006790	5		
sulfur compound transport	GO:0072348	2		
transcription regulator activity	GO:0030528			2

Table S7 GO enrichment analysis of genes significantly regulated in response to *FLO11* over-expression in ∑1278b (indicated as Σ1278b *FLO11*) using the online application FunSpec (p<0.0001; (Robinson *et al.* 2002). Shown is the number of genes identified to group in each category.

GO Term	GO ID	Sigma *FLO11+*
branched chain family amino acid catabolic process	GO:0009083	4
catalytic activity	GO:0003824	24
cellular aromatic compound metabolic process	GO:0006725	8
metabolic process	GO:0008152	21
organic acid metabolic process	GO:0006082	9
response to temperature stimulus	GO:0009266	5
transmembrane transport	GO:0055085	22
transporter activity	GO:0005215	13

REFERENCES

Bester, M. C., I. S. Pretorius and F. F. Bauer, 2006 The regulation of Saccharomyces cerevisiae *FLO* gene expression and Ca^{2+}-dependent flocculation by Flo8p and Mss11p. Curr Genet **49:** 375-383.

Brachmann, C. B., A. Davies, G. J. Cost, E. Caputo, J. Li *et al.*, 1998 Designer deletion strains derived from *Saccharomyces cerevisiae* S288C: a useful set of strains and plasmids for PCR-mediated gene disruption and other applications. Yeast **14:** 115-132.

Gagiano, M., D. Van Dyk, F. F. Bauer, M. G. Lambrechts and I. S. Pretorius, 1999a Divergent regulation of the evolutionarily closely related promoters of the *Saccharomyces cerevisiae STA2* and *MUC1* genes. J Bacteriol **181:** 6497-6508.

Gagiano, M., D. van Dyk, F. F. Bauer, M. G. Lambrechts and I. S. Pretorius, 1999b Msn1p/Mss10p, Mss11p and Muc1p/Flo11p are part of a signal transduction pathway downstream of Mep2p regulating invasive growth and pseudohyphal differentiation in *Saccharomyces cerevisiae*. Mol Microbiol **31:** 103-116.

Gietz, R. D., and A. Sugino, 1988 New yeast-*Escherichia coli* shuttle vectors constructed with in vitro mutagenized yeast genes lacking six-base pair restriction sites. Gene **74:** 527-534.

Robinson, M. D., J. Grigull, N. Mohammad and T. R. Hughes, 2002 FunSpec: a web-based cluster interpreter for yeast. BMC Bioinformatics **3:** 35.

van Dyk, D., I. S. Pretorius and F. F. Bauer, 2005 Mss11p is a central element of the regulatory network that controls *FLO11* expression and invasive growth in *Saccharomyces cerevisiae*. Genetics **169:** 91-106.

Verstrepen, K. J., A. Jansen, F. Lewitter and G. R. Fink, 2005 Intragenic tandem repeats generate functional variability. Nat Genet **37:** 986-990.
