# Supplementary material for: Many Saccharomyces cerevisiae Cell Wall Protein Encoding Genes Are Coregulated by Mss11, but Cellular Adhesion Phenotypes Appear Only Flo Protein Dependent
Source: G3 (Bethesda). 2012 Jan 1;2(1):131–41. doi: 10.1534/g3.111.001644 (PMC3276193; doi:10.1534/g3.111.001644)
Supplement: Supporting Information [file supp_2.1.131_FigureS1.pdf]

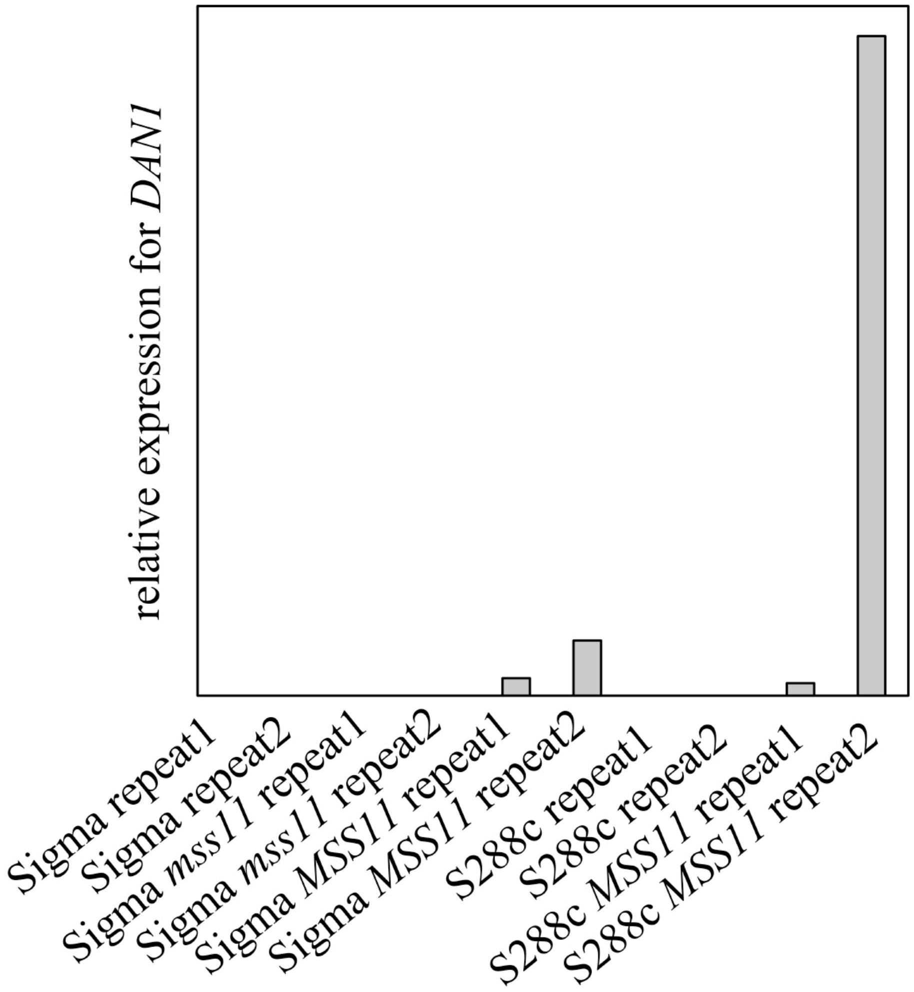

Figure S1 *DAN1* transcript is only detected in strains over-expressing *MSS11* as determined by qPCR. Shown is the relative expression data of each individual repeat for Σ1278b and S288c wild type strains (labeled Sigma and S288c respectively), Σ1278b with a *MSS11* deletion (Sigma *mss11*) and strains over-expressing *MSS11* (Sigma *MSS11* and S288c *MSS11*). Except for the over-expression strains no signal could be detected corresponding to *DAN1* transcript.
