# Supplementary material for: Many Saccharomyces cerevisiae Cell Wall Protein Encoding Genes Are Coregulated by Mss11, but Cellular Adhesion Phenotypes Appear Only Flo Protein Dependent
Source: G3 (Bethesda). 2012 Jan 1;2(1):131–41. doi: 10.1534/g3.111.001644 (PMC3276193; doi:10.1534/g3.111.001644)
Supplement: Supporting Information [file supp_2.1.131_FigureS2.pdf]

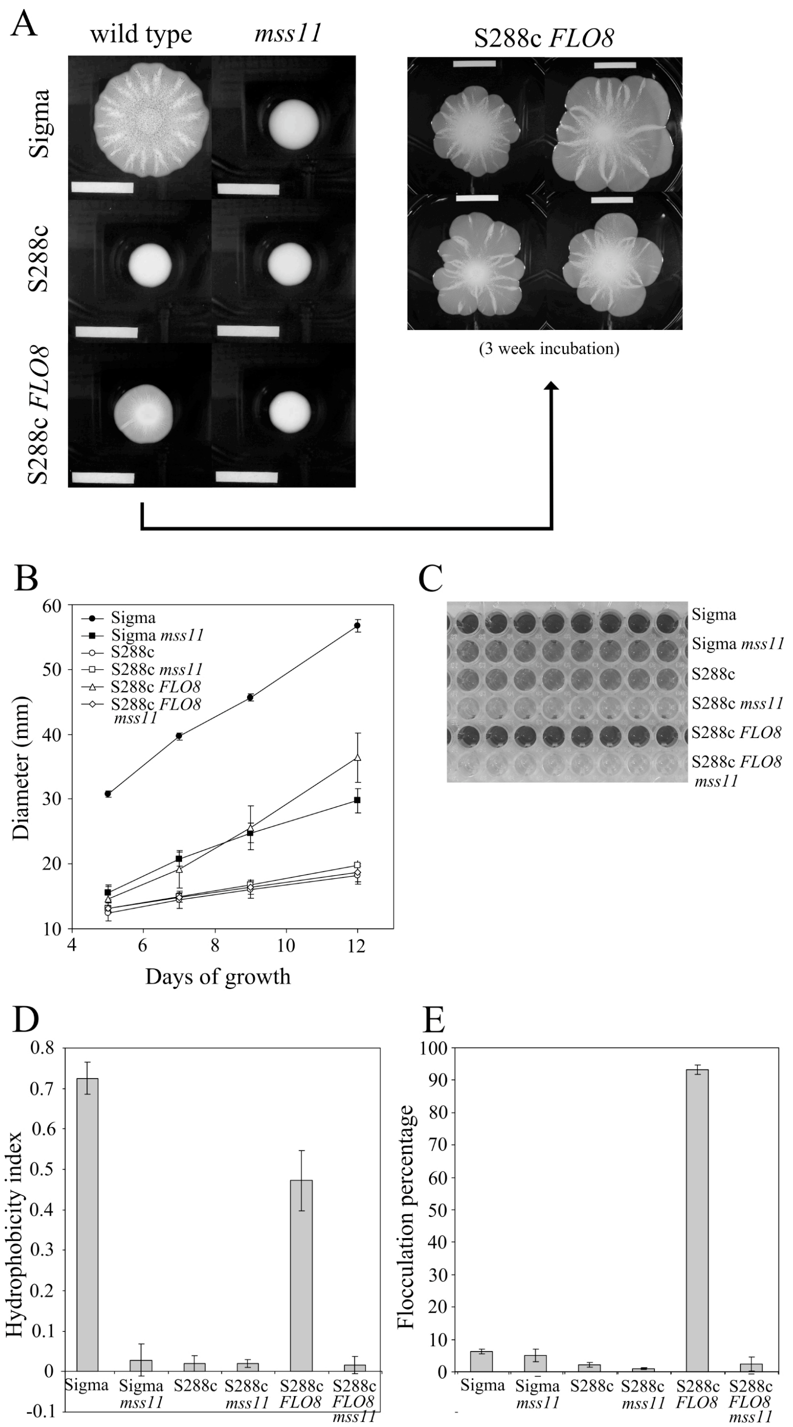

Figure S2 Adhesion phenotype analysis of strains Σ1278b (labeled "Sigma"), S288c and S288c (FLO8) respectively. Various cell-cell and cell-substrate interactions were investigated for wild type and MSS11 deletion (mss11) strains. (A) “Mat” formation on 0.3% YPD agar after 9 days of growth (left panel). Strain S288c (FLO8) was further incubated for ~2 weeks to allow for fully developed “mats” (right panel). Growth was measured up to 12 days post seeding as described in Materials and Methods (B). Strains grown to stationary phase in YPD were assayed for their ability to adhere to polystyrene (C), their degree of hydrophobicity (D) and for flocculation (E).
