# Supplementary material for: Many Saccharomyces cerevisiae Cell Wall Protein Encoding Genes Are Coregulated by Mss11, but Cellular Adhesion Phenotypes Appear Only Flo Protein Dependent
Source: G3 (Bethesda). 2012 Jan 1;2(1):131–41. doi: 10.1534/g3.111.001644 (PMC3276193; doi:10.1534/g3.111.001644)
Supplement: Supporting Information [file supp_2.1.131_FigureS3.pdf]

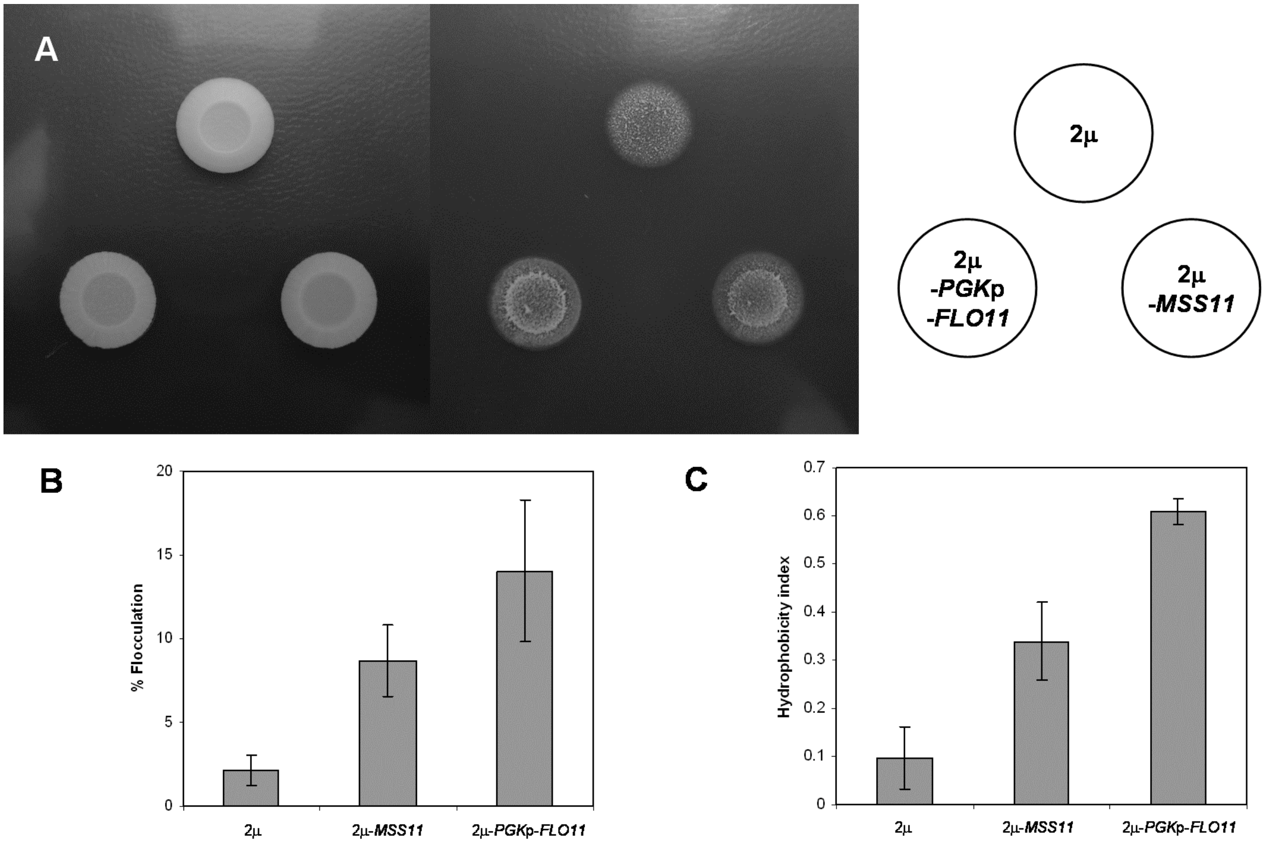

Figure S3 Adhesion phenotypes of strains either over-expressing *FLO11* or *MSS11*. Shown is (A) the ability to invade SCD agar plates following incubation of 12 days, (B) the degree of flocculent behavior and (C) outer cell hydrophobicity of transformants grown to stationary growth phase.
