# Supplementary material for: Many Saccharomyces cerevisiae Cell Wall Protein Encoding Genes Are Coregulated by Mss11, but Cellular Adhesion Phenotypes Appear Only Flo Protein Dependent
Source: G3 (Bethesda). 2012 Jan 1;2(1):131–41. doi: 10.1534/g3.111.001644 (PMC3276193; doi:10.1534/g3.111.001644)
Supplement: Supporting Information [file supp_2.1.131_FigureS4.pdf]

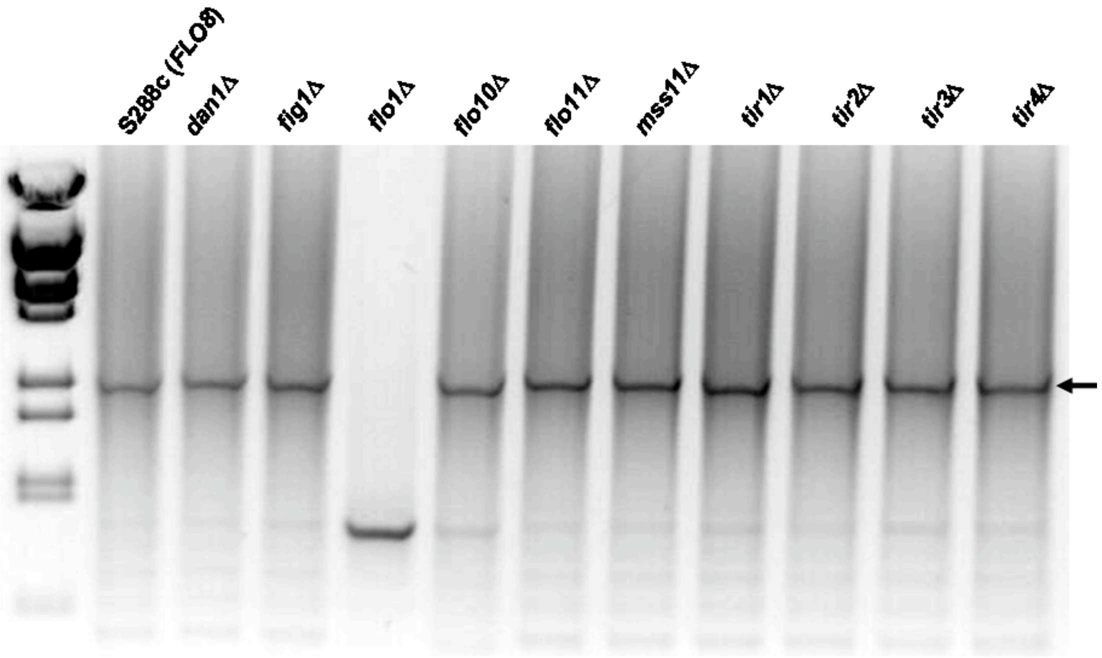

Figure S4 *FLO1* intragenic tandem repeat analysis showing repeats are of similar size in the single deletion strain set. *FLO1* repeats were PCR amplified using primers as described before (Verstrepen *et al.* 2005). DNA marker is lamda DNA digested with the restriction endo-nuclease *Bst*EII.
