# Supplementary material for: Many Saccharomyces cerevisiae Cell Wall Protein Encoding Genes Are Coregulated by Mss11, but Cellular Adhesion Phenotypes Appear Only Flo Protein Dependent
Source: G3 (Bethesda). 2012 Jan 1;2(1):131–41. doi: 10.1534/g3.111.001644 (PMC3276193; doi:10.1534/g3.111.001644)
Supplement: Supporting Information [file supp_2.1.131_TableS1.pdf]

Table S1 Plasmids used in this study

Plasmid name	Relevant genotype	Source or reference
YEpLac181	2μ *LEU2*	(Gietz and Sugino 1988)
YEpLac195	2μ *URA3*	(Gietz and Sugino 1988)
YEpLac181-MSS11	2μ *LEU2 MSS11*	(Gagiano *et al.* 1999a)
YEpLac181-PGKp-MUC1	2μ *LEU2 PGK1*promoter *FLO11*	this lab
YEpLac195-MSS11	2μ *URA3 MSS11*	(Gagiano *et al.* 1999b)
