# Supplementary material for: Many Saccharomyces cerevisiae Cell Wall Protein Encoding Genes Are Coregulated by Mss11, but Cellular Adhesion Phenotypes Appear Only Flo Protein Dependent
Source: G3 (Bethesda). 2012 Jan 1;2(1):131–41. doi: 10.1534/g3.111.001644 (PMC3276193; doi:10.1534/g3.111.001644)
Supplement: Supporting Information [file supp_2.1.131_TableS2.pdf]

Table 2 ***S. cerevisiae*** **strains used in this study**

Strain	Relevant genotype	Source or reference
BY4742	*MATα his3*Δ1 *leu2*Δ0 *lys2*Δ0 *ura3*Δ0	(Brachmann *et al.* 1998)
BY4742 *dan1*Δ	isogenic to BY4742 *dan1*Δ::*KanMX4*	EUROSCARF
BY4742 *fig1*Δ	isogenic to BY4742 *fig1*Δ::*KanMX4*	EUROSCARF
BY4742 *fig2*Δ	isogenic to BY4742 *fig2*Δ::*KanMX4*	EUROSCARF
BY4742 *flo1*Δ	isogenic to BY4742 *flo1*Δ::*KanMX4*	EUROSCARF
BY4742 *flo10*Δ	isogenic to BY4742 *flo10*Δ::*KanMX4*	EUROSCARF
BY4742 *mss11*Δ	isogenic to BY4742 *mss11*Δ::*KanMX4*	EUROSCARF
BY4742 *nca3*Δ	isogenic to BY4742 *nca3*Δ::*KanMX4*	EUROSCARF
BY4742 *tir1*Δ	isogenic to BY4742 *tir1*Δ::*KanMX4*	EUROSCARF
BY4742 *tir2*Δ	isogenic to BY4742 *tir2*Δ::*KanMX4*	EUROSCARF
BY4742 *tir3*Δ	isogenic to BY4742 *tir3*Δ::*KanMX4*	EUROSCARF
BY4742 *tir4*Δ	isogenic to BY4742 *tir4*Δ::*KanMX4*	EUROSCARF
BY4742 *FLO8*	isogenic to BY4742 *flo8-1*Δ::*FLO8-LEU2*	(Bester *et al.* 2006)
BY4742 *FLO8 dan1*Δ	isogenic to BY4742 *dan1*Δ::*KanMX4 flo8-1*Δ::*FLO8-LEU2*	This study
BY4742 *FLO8 fig1*Δ	isogenic to BY4742 *fig1*Δ::*KanMX4 flo8-1*Δ::*FLO8-LEU2*	This study
BY4742 *FLO8 flo1*Δ	isogenic to BY4742 *flo8-1*Δ::*FLO8-LEU2 flo1*Δ::*KanMX4*	(Bester *et al.* 2006)
BY4742 *FLO8 flo10*Δ	isogenic to BY4742 *flo8-1*Δ::*FLO8-LEU2 flo10*Δ::*KanMX4*	(Bester *et al.* 2006)
BY4742 *FLO8 flo11*Δ	isogenic to BY4742 *flo8-1*Δ::*FLO8-LEU2 flo11*Δ::*lacZ-HIS3*	(Bester *et al.* 2006)
BY4742 *FLO8 tir1*Δ	isogenic to BY4742 *tir1*Δ::*KanMX4 flo8-1*Δ::*FLO8-LEU2*	This study
BY4742 *FLO8 tir2*Δ	isogenic to BY4742 *tir2*Δ::*KanMX4 flo8-1*Δ::*FLO8-LEU2*	This study
BY4742 *FLO8 tir3*Δ	isogenic to BY4742 *tir3*Δ::*KanMX4 flo8-1*Δ::*FLO8-LEU2*	This study

BY4742 *FLO8 tir4Δ*	isogenic to BY4742 *tir4Δ::KanMX4 flo8-1Δ::FLO8-LEU2*	This study
BY4742 *FLO8 dan1Δ flo11Δ::lacZ*	isogenic to BY4742 *dan1Δ::KanMX4 flo8-1Δ::FLO8-LEU2 flo11Δ::lacZ-HIS3*	This study
BY4742 *FLO8 fig1Δ flo11Δ::lacZ*	isogenic to BY4742 *fig1Δ::KanMX4 flo8-1Δ::FLO8-LEU2 flo11Δ::lacZ-HIS3*	This study
BY4742 *FLO8 fig2Δ flo11Δ::lacZ*	isogenic to BY4742 *fig2Δ::KanMX4 flo8-1Δ::FLO8-LEU2 flo11Δ::lacZ-HIS3*	This study
BY4742 *FLO8 flo1Δ flo11Δ::lacZ*	isogenic to BY4742 *flo1Δ::KanMX4 flo8-1Δ::FLO8-LEU2 flo11Δ::lacZ-HIS3*	This study
BY4742 *FLO8 flo10Δ flo11Δ::lacZ*	isogenic to BY4742 *flo10Δ::KanMX4 flo8-1Δ::FLO8-LEU2 flo11Δ::lacZ-HIS3*	This study
BY4742 *FLO8 flo11Δ::lacZ nca3Δ*	isogenic to BY4742 *nca3Δ::KanMX4 flo8-1Δ::FLO8-LEU2 flo11Δ::lacZ-HIS3*	This study
BY4742 *FLO8 flo11Δ::lacZ tir1Δ*	isogenic to BY4742 *tir1Δ::KanMX4 flo8-1Δ::FLO8-LEU2 flo11Δ::lacZ-HIS3*	This study
BY4742 *FLO8 flo11Δ::lacZ tir2Δ*	isogenic to BY4742 *tir2Δ::KanMX4 flo8-1Δ::FLO8-LEU2 flo11Δ::lacZ-HIS3*	This study
BY4742 *FLO8 flo11Δ::lacZ tir3Δ*	isogenic to BY4742 *tir3Δ::KanMX4 flo8-1Δ::FLO8-LEU2 flo11Δ::lacZ-HIS3*	This study
BY4742 *FLO8 flo11Δ::lacZ tir4Δ*	isogenic to BY4742 *tir4Δ::KanMX4 flo8-1Δ::FLO8-LEU2 flo11Δ::lacZ-HIS3*	This study
YHUM272	*MATα ura3-52 trp1Δ::hisG leu2Δ::hisG his3Δ::hisG*	H.-U. Mösch
Σ1278b *flo8*	isogenic to Σ1278b *flo8Δ::LEU2*	(van Dyk et al. 2005)
Σ1278b *flo11*	isogenic to Σ1278b *flo11Δ::lacZ-HIS3*	(van Dyk et al. 2005)
Σ1278b *mss11*	isogenic to Σ1278b *mss11Δ::LEU2*	(van Dyk et al. 2005)
Σ1278b *sfl1*	isogenic to Σ1278b *sfl1Δ::KanMX4*	(van Dyk et al. 2005)
Σ1278b *ste12*	isogenic to Σ1278b *ste12Δ::URA3*	(van Dyk et al. 2005)
Σ1278b *tec1*	isogenic to Σ1278b *tec1Δ::LEU2*	(van Dyk et al. 2005)
Σ1278b *sfl1 flo8*	isogenic to Σ1278b *sfl1Δ::KanMX4 flo8Δ::LEU2*	(van Dyk et al. 2005)
Σ1278b *sfl1 mss11*	isogenic to Σ1278b *sfl1Δ::KanMX4 mss11Δ::LEU2*	(van Dyk et al. 2005)
Σ1278b *sfl1 ste12*	isogenic to Σ1278b *sfl1Δ::KanMX4 ste12Δ::URA3*	(van Dyk et al. 2005)
Σ1278b *sfl1 tec1*	isogenic to Σ1278b *sfl1Δ::KanMX4 tec1Δ::LEU2*	(van Dyk et al. 2005)
Σ1278b *dan1Δ flo11Δ::lacZ*	isogenic to Σ1278b *dan1Δ::KanMX4 flo11Δ::lacZ-HIS3*	This study
Σ1278b *fig1Δ flo11Δ::lacZ*	isogenic to Σ1278b *figΔ::KanMX4 flo11Δ::lacZ-HIS3*	This study

Σ1278b *fig2*Δ *flo11*Δ::*lacZ*	isogenic to Σ1278b *fig2*Δ::*KanMX4 flo11*Δ::*lacZ-HIS3*	This study
Σ1278b *flo1*Δ *flo11*Δ::*lacZ*	isogenic to Σ1278b *flo1*Δ::*KanMX4 flo11*Δ::*lacZ-HIS3*	This study
Σ1278b *flo10*Δ *flo11*Δ::*lacZ*	isogenic to Σ1278b *flo10*Δ::*KanMX4 flo11*Δ::*lacZ-HIS3*	This study
Σ1278b *flo11*Δ::*lacZ nca3*Δ	isogenic to Σ1278b *nca3*Δ::*KanMX4 flo11*Δ::*lacZ-HIS3*	This study
Σ1278b *flo11*Δ::*lacZ tir1*Δ	isogenic to Σ1278b *tir1*Δ::*KanMX4 flo11*Δ::*lacZ-HIS3*	This study
Σ1278b *flo11*Δ::*lacZ tir2*Δ	isogenic to Σ1278b *tir2*Δ::*KanMX4 flo11*Δ::*lacZ-HIS3*	This study
Σ1278b *flo11*Δ::*lacZ tir3*Δ	isogenic to Σ1278b *tir3*Δ::*KanMX4 flo11*Δ::*lacZ-HIS3*	This study
Σ1278b *flo11*Δ::*lacZ tir4*Δ	isogenic to Σ1278b *tir4*Δ::*KanMX4 flo11*Δ::*lacZ-HIS3*	This study
