# Supplementary material for: Many Saccharomyces cerevisiae Cell Wall Protein Encoding Genes Are Coregulated by Mss11, but Cellular Adhesion Phenotypes Appear Only Flo Protein Dependent
Source: G3 (Bethesda). 2012 Jan 1;2(1):131–41. doi: 10.1534/g3.111.001644 (PMC3276193; doi:10.1534/g3.111.001644)
Supplement: Supporting Information [file supp_2.1.131_TableS3.pdf]

Table S3 Primers used for the PCR amplification of gene disruption cassettes

Primer name	Primer sequence (5'→3')
AQY2kanMX4-F	CTTTCATTAACGAATTAGAGCGC
AQY2kanMX4-R	TCAAAGCCATGTGAGCCATG
DAN1kanMX4-F	CTCCGTAGACGCTCCTCTGAA
DAN1kanMX4-R	TGTTAAGCTGTCTGCAATAAGGAAT
FIG1kanMX4-F	GATGGTTTCATGTATGTGTCAGTTAAA
FIG1kanMX4-R	GTCGCTCATCAAGGTGACAGTAA
FIG2kanMX4-F	GGTACATGGTGCTTTCCTTATGC
FIG2kanMX4-R	GCCTTGATTGCAGAGGTTGTG
FLO1-Fp-Conf	CGATAGGGAGGCATCATGGTACTACCG
FLO1-Rp-Conf	AAGAAGCGCAAGAATTATCATTTAGTCAAT
FLO10-F	AAATGGGCTCCTGCCTGAAT
FLO10-R	CTAGCTCATCCGTTGCCGCT
HPF1(YOL155c)kanMX4-F	GCAGAAGTGCCCGTAGGAGA
HPF1(YOL155c)kanMX4-R	GTGGACAATAAGTGAAATAAGTGTCCTT
NCA3kanMX4-F	GTGGCAAAGCGGACAGCT
NCA3kanMX4-R	CCGTAATGCAGAGTACACCTTGA
TIR1kanMX4-F	GAACTGCGTTTGTATGCAACTGT
TIR1kanMX4-R	GTATCCAACAGACAGTAGTGCCAAC
tir2kof	GCTGAGCATCATGTGAGTAA
tir2kor	ATGTGGGCAGGAAGACATGC
tir3kof	GCCCTAGAATGGATTGCAGA
tir3kor	TGCTCTTGCTCGAACATTCC
TIR4kanMX4-F	GCATTTCTAACAAGTAGGATAGTCCAA
TIR4kanMX4-R	GATGATGCTAGGATAGGCACCTT
