# Supplementary material for: Many Saccharomyces cerevisiae Cell Wall Protein Encoding Genes Are Coregulated by Mss11, but Cellular Adhesion Phenotypes Appear Only Flo Protein Dependent
Source: G3 (Bethesda). 2012 Jan 1;2(1):131–41. doi: 10.1534/g3.111.001644 (PMC3276193; doi:10.1534/g3.111.001644)
Supplement: Supporting Information [file supp_2.1.131_TableS4.pdf]

Table S4 Primers and hydrolysis probes used for qPCR analysis

Primer/probe name	Primer/probe sequence (5’→3’)	Modifications
FLO9-F (TaqmanMGB)	TGTACAATAAAAGCCCCAAAAATG	none
FLO9-R (TaqmanMGB)	GCAATGTGACGATGGCTAGTAGTAA	none
FLO9-probe	CTCTGGCACATTATT	NED dye (5'), Minor Groove Binder (3')
AQY2-F-(rt-PCR)	GGACCCGACCGGTGTTG	none
AQY2-R-(rt-PCR)	TTAAAACGCGAATGCTTCGTT	none
DAN1-F-(rt-PCR)	GCTTTCCAGGCTTTGCATAAGA	none
DAN1-R-(rt-PCR)	TCGCCACCGGCAAAAA	none
DAN4-F-(rt-PCR)	GCCACTACATCGAACAATGCA	none
DAN4-R-(rt-PCR)	GGCACCCGCAGAGCAA	none
FIG1-F-(rt-PCR)	TCCCTTATACAGAGACTTGGAAATTCA	none
FIG1-R-(rt-PCR)	AATTGGGCTAACTTCAAAATGTTCA	none
FIG2-F-(rt-PCR)	CTTCTGATACTTTTTCTTCATACTCTGATATCT	none
FIG2-R-(rt-PCR)	TGTCCTATGAGGTTGTGCAGTTG	none
FLO11-F-(QRT-PCR)	CCTCCGAAGGAACTAGCTGTAATT	none
FLO11-R-(QRT-PCR)	AGTCACATCCAAAGTATACTGCATGAT	none
HPF1(YIL169c)-F-(rt-PCR)	CTAAGGACATACACTACTGCCACTGGT	none
HPF1(YIL169c)-R-(rt-PCR)	ACTAGTTGCGTGACGGTTGAAGTAG	none
HPF1(YOL155c)-F-(rt-PCR)	CGGTTCATCTTCTGCCACAGA	none
HPF1(YOL155c)-R-(rt-PCR)	GTTCATCTTCTGCCACAGAATCAG	none
NCA3-F-(rt-PCR)	TGGTGGATGGGCCTCTGT	none
NCA3-R-(rt-PCR)	GACATTCCAGGTTCACATGCA	none
PDA1-F-QRT-PCR	GGAATTTGCCCGTCGTGTT	none
PDA1-R-QRT-PCR	GCGGCGGTACCCATACC	none
TIR1-F-(rt-PCR)	TCCAAGCTACCAAGGCTGTTTC	none
TIR1-R-(rt-PCR)	ACCCATACCAACAAAGGCCTTA	none
TIR2-F-qPCR	CTGCCCAAACTCAGGAAGAAA	none
TIR2-R-qPCR	CTTGCAAGTTGGACTTAACGTCAT	none
TIR3-F-(QRT-PCR)	TTTGACGCTATTTTGGCTGATG	none

TIR3-R-(QRT-PCR)	TCTGGATTATTCATTGCCAAGGA	none
TIR4-F-(rt-PCR)	TGCCGACTACATCACCCTATCC	none
TIR4-R-(rt-PCR)	GGCATTTGGTCCAAGGAAAA	none
