# Supplementary material for: Many Saccharomyces cerevisiae Cell Wall Protein Encoding Genes Are Coregulated by Mss11, but Cellular Adhesion Phenotypes Appear Only Flo Protein Dependent
Source: G3 (Bethesda). 2012 Jan 1;2(1):131–41. doi: 10.1534/g3.111.001644 (PMC3276193; doi:10.1534/g3.111.001644)
Supplement: Supporting Information [file supp_2.1.131_TableS5.pdf]

**Table S5 Genes significantly regulated in response to *MSS11* over-expression or deletion in ∑1278b (indicated as Σ1278b *MSS11* and Σ1278b *mss11* respectively) or over-expression in S288c (S288c *MSS11*)**. Shown are the degree of regulation ($log_2$fold), the open reading frame (ORF), the gene name if applicable as well as a short description as obtained from the *Saccharomyces genome database* (SGD).

| $log_2$fold | ORF | Gene name | Short description |
|---|---|---|---|
| S288c *MSS11* | | | |
| -2.738 | YJL170C | *ASG7* | Protein that regulates signaling from a G protein beta subunit Ste4p and its relocalization within the cell |
| -2.307 | YIL015W | *BAR1* | Aspartyl protease secreted into the periplasmic space of mating type a cells, helps cells find mating partners, cleaves and inactivates alpha factor allowing cells to recover from alpha-factor-induced cell cycle arrest |
| -1.87 | YCL026C-A | *FRM2* | Protein of unknown function, involved in the integration of lipid signaling pathways with cellular homeostasis |
| -1.148 | YMR279C | | Putative protein of unknown function |
| -1.066 | YJL160C | | Putative protein of unknown function |
| -1.061 | YDL127W | *PCL2* | G1 cyclin, associates with Pho85p cyclin-dependent kinase (Cdk) to contribute to entry into the mitotic cell cycle, essential for cell morphogenesis |
| -0.919 | YBR019C | *GAL10* | UDP-glucose-4-epimerase, catalyzes the interconversion of UDP-galactose and UDP-D-glucose in galactose metabolism |
| -0.919 | YOR114W | | Putative protein of unknown function |
| -0.887 | YPL130W | *SPO19* | Meiosis-specific protein of unknown function, involved in completion of nuclear divisions |
| -0.85 | R0020C | *REP1* | Master regulator that acts in concert with Rep2p to regulate transcript levels of the FLP1 gene that promotes plasmid copy amplification |
| -0.849 | YGR131W | | Protein of unknown function |
| -0.807 | YDR106W | *ARP10* | Component of the dynactin complex, localized to the pointed end of the Arp1p filament |
| -0.7 | YJR112W | *NNF1* | Essential component of the MIND kinetochore complex (Mtw1p Including Nnf1p-Nsl1p-Dsn1p) which joins kinetochore subunits contacting DNA to those contacting microtubules |
| -0.655 | YPR106W | *ISR1* | Predicted protein kinase, overexpression causes sensitivity to staurosporine, which is a potent inhibitor of protein kinase C |

| | | | |
|---|---|---|---|
| -0.651 | YKL089W | *MIF2* | Kinetochore protein with homology to human CENP-C, required for structural integrity of the spindle during anaphase spindle elongation, interacts with histones H2A, H2B, and H4, phosphorylated by Ipl1p |
| -0.647 | YJL196C | *ELO1* | Elongase I, medium-chain acyl elongase, catalyzes carboxy-terminal elongation of unsaturated C12-C16 fatty acyl-CoAs to C16-C18 fatty acids |
| -0.627 | YDR179W-A | | Putative protein of unknown function |
| -0.626 | YHR063C | *PAN5* | 2-dehydropantoate 2-reductase, part of the pantothenic acid pathway, structurally homologous to E. coli panE |
| -0.569 | YFL046W | *FMP32* | Putative protein of unknown function |
| -0.513 | YML023C | *NSE5* | Essential subunit of the Mms21-Smc5-Smc6 complex |
| 0.5 | YNR018W | | Putative protein of unknown function |
| 0.746 | YLR216C | *CPR6* | Peptidyl-prolyl cis-trans isomerase (cyclophilin), catalyzes the cis-trans isomerization of peptide bonds N-terminal to proline residues |
| 0.783 | YHR112C | | Putative protein of unknown function |
| 0.859 | YPL024W | *RMI1* | Subunit of the RecQ (Sgs1p) - Topo III (Top3p) complex |
| 0.904 | YNL191W | *DUG3* | Probable glutamine amidotransferase, forms a complex with Dug1p and Dug2p to degrade glutathione (GSH) and other peptides containing a gamma-glu-X bond in an alternative pathway to GSH degradation by gamma-glutamyl transpeptidase (Ecm38p) |
| 0.966 | YER042W | *MXR1* | Methionine-S-sulfoxide reductase, involved in the response to oxidative stress |
| 1.039 | YGL010W | | Putative protein of unknown function |
| 1.162 | YPR196W | | Putative maltose activator |
| 1.225 | YAL012W | *CYS3* | Cystathionine gamma-lyase, catalyzes one of the two reactions involved in the transsulfuration pathway that yields cysteine from homocysteine with the intermediary formation of cystathionine |
| 1.249 | YNL276C | | |
| 1.249 | YNL277W | *MET2* | L-homoserine-O-acetyltransferase, catalyzes the conversion of homoserine to O-acetyl homoserine which is the first step of the methionine biosynthetic pathway |

| | | | |
|---|---|---|---|
| 1.465 | YDL059C | *RAD59* | Protein involved in the repair of double-strand breaks in DNA during vegetative growth via recombination and single-strand annealing |
| 1.501 | YDR044W | *HEM13* | Coproporphyrinogen III oxidase, an oxygen requiring enzyme that catalyzes the sixth step in the heme biosynthetic pathway |
| 1.521 | YHL036W | *MUP3* | Low affinity methionine permease, similar to Mup1p |
| 1.528 | YKL109W | *HAP4* | Subunit of the heme-activated, glucose-repressed Hap2p/3p/4p/5p CCAAT-binding complex, a transcriptional activator and global regulator of respiratory gene expression |
| 1.599 | YDL245C | *HXT15* | Protein of unknown function with similarity to hexose transporter family members, expression is induced by low levels of glucose and repressed by high levels of glucose |
| 1.599 | YJR158W | *HXT16* | Protein of unknown function with similarity to hexose transporter family members, expression is repressed by high levels of glucose |
| 1.655 | YOR009W | *TIR4* | Cell wall mannoprotein of the Srp1p/Tip1p family of serine-alanine-rich proteins |
| 1.658 | YLL061W | *MMP1* | High-affinity S-methylmethionine permease, required for utilization of S-methylmethionine as a sulfur source |
| 1.719 | YKL201C | *MNN4* | Putative positive regulator of mannosylphosphate transferase (Mn6p), involved in mannosylphosphorylation of N-linked oligosaccharides |
| 1.719 | YKL202W | | |
| 1.755 | YMR244W | | Putative protein of unknown function |
| 1.808 | YLR364W | *GRX8* | Glutaredoxin that employs a dithiol mechanism of catalysis |
| 1.881 | YDR253C | *MET32* | Zinc-finger DNA-binding protein, involved in transcriptional regulation of the methionine biosynthetic genes, similar to Met31p |
| 1.898 | YMR081C | *ISF1* | Serine-rich, hydrophilic protein with similarity to Mbr1p |
| 1.931 | YJL212C | *OPT1* | Proton-coupled oligopeptide transporter of the plasma membrane |
| 1.961 | YFL051C | | Putative protein of unknown function |
| 1.968 | YEL070W | *DSF1* | Deletion suppressor of mpt5 mutation |
| 1.968 | YNR073C | | Putative mannitol dehydrogenase |
| 2.1 | YGR055W | *MUP1* | High affinity methionine permease, integral membrane protein with 13 putative membrane-spanning regions |

| | | | |
|---|---|---|---|
| 2.374 | YAR064W | | Putative protein of unknown function |
| 2.374 | YHR213W-B | | Putative protein of unknown function |
| 2.458 | YER011W | *TIR1* | Cell wall mannoprotein of the Srp1p/Tip1p family of serine-alanine-rich proteins |
| 2.47 | YDR342C | *HXT7* | High-affinity glucose transporter of the major facilitator superfamily, nearly identical to Hxt6p, expressed at high basal levels relative to other HXTs, expression repressed by high glucose levels |
| 2.47 | YDR343C | *HXT6* | High-affinity glucose transporter of the major facilitator superfamily, nearly identical to Hxt7p, expressed at high basal levels relative to other HXTs, repression of expression by high glucose requires SNF3 |
| 2.522 | YKL068W-A | | Putative protein of unknown function |
| 2.629 | YJL218W | | Putative protein of unknown function, similar to bacterial galactoside O-acetyltransferases |
| 2.951 | YAL063C | *FLO9* | Lectin-like protein with similarity to Flo1p, thought to be expressed and involved in flocculation |
| 2.951 | YAL064W-B | | Fungal-specific protein of unknown function |
| 2.951 | YAR050W | *FLO1* | Lectin-like protein involved in flocculation, cell wall protein that binds to mannose chains on the surface of other cells, confers floc-forming ability that is chymotrypsin sensitive and heat resistant |
| 2.951 | YHR211W | *FLO5* | Lectin-like cell wall protein (flocculin) involved in flocculation, binds to mannose chains on the surface of other cells, confers floc-forming ability that is chymotrypsin resistant but heat labile |
| 2.966 | YHR092C | *HXT4* | High-affinity glucose transporter of the major facilitator superfamily, expression is induced by low levels of glucose and repressed by high levels of glucose |
| 3.052 | YMR317W | | Putative protein of unknown function with some similarity to sialidase from Trypanosoma |
| 3.172 | YMR164C | *MSS11* | Transcription factor involved in regulation of invasive growth and starch degradation |
| 3.214 | YDL039C | *PRM7* | Pheromone-regulated protein, predicted to have one transmembrane segment |
| 3.23 | YIL011W | *TIR3* | Cell wall mannoprotein of the Srp1p/Tip1p family of serine-alanine-rich proteins |
| 3.319 | YMR011W | *HXT2* | High-affinity glucose transporter of the major facilitator superfamily, expression is induced by low levels of glucose and repressed by |

| | | | |
|---|---|---|---|
| | | | high levels of glucose |
| 3.732 | YDL039C | *PRM7* | Pheromone-regulated protein, predicted to have one transmembrane segment |
| 3.747 | YAL065C | | Putative protein of unknown function |
| 3.822 | YOR010C | *TIR2* | Putative cell wall mannoprotein of the Srp1p/Tip1p family of serine-alanine-rich proteins |
| 3.853 | YJR150C | *DAN1* | Cell wall mannoprotein with similarity to Tir1p, Tir2p, Tir3p, and Tir4p |
| 3.876 | YHR213W-A | | Putative protein of unknown function |
| 4.818 | YJL116C | *NCA3* | Protein that functions with Nca2p to regulate mitochondrial expression of subunits 6 (Atp6p) and 8 (Atp8p ) of the Fo-F1 ATP synthase |
| 4.991 | YIR019C | *MUC1* | GPI-anchored cell surface glycoprotein (flocculin) required for pseudohyphal formation, invasive growth, flocculation, and biofilms |
| 5.373 | YAR062W | | Hypothetical protein |
| 5.373 | YHR213W | | Putative protein of unknown function |
| 5.798 | YAR050W | *FLO1* | Lectin-like protein involved in flocculation, cell wall protein that binds to mannose chains on the surface of other cells, confers floc-forming ability that is chymotrypsin sensitive and heat resistant |
| Σ1278b *MSS11* | | | |
| -0.857 | YOL147C | *PEX11* | Peroxisomal membrane protein required for peroxisome proliferation and medium-chain fatty acid oxidation, most abundant protein in the peroxisomal membrane, regulated by Adr1p and Pip2p-Oaf1p, promoter contains ORE and UAS1-like elements |
| -0.855 | YHR143W | *DSE2* | Daughter cell-specific secreted protein with similarity to glucanases, degrades cell wall from the daughter side causing daughter to separate from mother |
| 0.916 | YNL036W | *NCE103* | Carbonic anhydrase |
| 1.086 | YGR213C | *RTA1* | Protein involved in 7-aminocholesterol resistance |
| 1.204 | YOR032C | *HMS1* | Basic helix-loop-helix (bHLH) protein with similarity to myc-family transcription factors |
| 1.321 | YMR317W | | Putative protein of unknown function with some similarity to sialidase from Trypanosoma |
| 1.321 | YIL013C | *PDR11* | ATP-binding cassette (ABC) transporter, multidrug transporter involved in multiple drug resistance |

| | | | |
|---|---|---|---|
| 1.403 | YHR213W-A | | Putative protein of unknown function |
| 1.437 | YAL063C | *FLO9* | Lectin-like protein with similarity to Flo1p, thought to be expressed and involved in flocculation |
| 1.437 | YAL064W-B | | Fungal-specific protein of unknown function |
| 1.437 | YAR050W | *FLO1* | Lectin-like protein involved in flocculation, cell wall protein that binds to mannose chains on the surface of other cells, confers floc-forming ability that is chymotrypsin sensitive and heat resistant |
| 1.437 | YHR211W | *FLO5* | Lectin-like cell wall protein (flocculin) involved in flocculation, binds to mannose chains on the surface of other cells, confers floc-forming ability that is chymotrypsin resistant but heat labile |
| 1.494 | YDL039C | *PRM7* | Pheromone-regulated protein, predicted to have one transmembrane segment |
| 1.528 | YNR002C | *ATO2* | Putative transmembrane protein involved in export of ammonia, a starvation signal that promotes cell death in aging colonies |
| 1.571 | YHL043W | *ECM34* | Putative protein of unknown function |
| 1.66 | YOR009W | *TIR4* | Cell wall mannoprotein of the Srp1p/Tip1p family of serine-alanine-rich proteins |
| 1.892 | YMR081C | *ISF1* | Serine-rich, hydrophilic protein with similarity to Mbr1p |
| 2.206 | YAR050W | *FLO1* | Lectin-like protein involved in flocculation, cell wall protein that binds to mannose chains on the surface of other cells, confers floc-forming ability that is chymotrypsin sensitive and heat resistant |
| 2.229 | YJL116C | *NCA3* | Protein that functions with Nca2p to regulate mitochondrial expression of subunits 6 (Atp6p) and 8 (Atp8p ) of the Fo-F1 ATP synthase |
| 2.452 | YAR062W | | Hypothetical protein |
| 2.452 | YHR213W | | Putative protein of unknown function |
| 2.49 | YIR019C | *MUC1* | GPI-anchored cell surface glycoprotein (flocculin) required for pseudohyphal formation, invasive growth, flocculation, and biofilms |
| 2.586 | YIL011W | *TIR3* | Cell wall mannoprotein of the Srp1p/Tip1p family of serine-alanine-rich proteins |
| 2.791 | YDL039C | *PRM7* | Pheromone-regulated protein, predicted to have one transmembrane segment |
| 4.329 | YMR164C | *MSS11* | Transcription factor involved in regulation of invasive growth and starch degradation |
| 4.497 | YOR010C | *TIR2* | Putative cell wall mannoprotein of the Srp1p/Tip1p family of serine-alanine-rich proteins |

| Σ1278b *mss11* | | | |
|---|---|---|---|
| -3.171 | YIR019C | *MUC1* | GPI-anchored cell surface glycoprotein (flocculin) required for pseudohyphal formation, invasive growth, flocculation, and biofilms |
| -2.962 | YMR164C | *MSS11* | Transcription factor involved in regulation of invasive growth and starch degradation |
| -2.426 | YOR032C | *HMS1* | Basic helix-loop-helix (bHLH) protein with similarity to myc-family transcription factors |
| -2.199 | YDR259C | *YAP6* | Putative basic leucine zipper (bZIP) transcription factor |
| -1.683 | YIL011W | *TIR3* | Cell wall mannoprotein of the Srp1p/Tip1p family of serine-alanine-rich proteins |
| -1.656 | YHL043W | *ECM34* | Putative protein of unknown function |
| -1.21 | YPR013C | | Putative zinc finger protein |
