# Supplementary material for: Many Saccharomyces cerevisiae Cell Wall Protein Encoding Genes Are Coregulated by Mss11, but Cellular Adhesion Phenotypes Appear Only Flo Protein Dependent
Source: G3 (Bethesda). 2012 Jan 1;2(1):131–41. doi: 10.1534/g3.111.001644 (PMC3276193; doi:10.1534/g3.111.001644)
Supplement: Supporting Information [file supp_2.1.131_TableS6.pdf]

Table S6 GO enrichment analysis of genes significantly regulated in response to *MSS11* over-expression or deletion in ∑1278b (indicated as Σ1278b *MSS11* and Σ1278b *mss11* respectively) or over-expression in S288c (S288c *MSS11*). Shown is the number of genes identified to group in each category as selected by the use of GOEast with a Benjamini & Yekutieli calculated FDR threshold of 0.1. Categories mentioned in text are shown in bold.

GO Term	GO ID	S288c *MSS11*	Sigma *MSS11*	Sigma *mss11*
DNA binding	GO:0003677			3
L-amino acid transmembrane transporter activity	GO:0015179	2		
amine transmembrane transporter activity	GO:0005275	3		
amino acid transmembrane transporter activity	GO:0015171	3		
amino acid transport	GO:0006865	3		
**anchored to membrane**	**GO:0031225**	**9**	**7**	**2**
carbohydrate binding	GO:0030246	3	2	
carbohydrate transmembrane transporter activity	GO:0015144	4		
carbohydrate transport	GO:0008643	4		
cell	GO:0005623			7
cell growth	GO:0016049		4	3
cell part	GO:0044464			7
**cell periphery**	**GO:0071944**	**16**	**8**	
**cell wall**	**GO:0005618**	**10**	**7**	**2**
cell wall organization	GO:0071555		3	
cellular cell wall organization	GO:0007047		3	
**external encapsulating structure**	**GO:0030312**	**10**	**7**	**2**
external encapsulating structure organization	GO:0045229		3	
**extracellular region**	**GO:0005576**	**11**	**7**	**2**
**filamentous growth**	**GO:0030447**		**4**	**3**
filamentous growth of a population of unicellular organisms	GO:0044182		4	3
**flocculation**	**GO:0000128**	**4**	**4**	**1**
flocculation via cell wall protein-carbohydrate interaction	GO:0000501	2	2	
fructose transmembrane transporter activity	GO:0005353	4		
**fungal-type cell wall**	**GO:0009277**	**7**	**5**	**1**
glucose transmembrane transporter activity	GO:0005355	4		

GO Term	GO ID	S288c *MSS11*	Sigma *MSS11*	Sigma *mss11*
growth	GO:0040007		4	3
growth of unicellular organism as a thread of attached cells	GO:0070783		4	3
hexose transmembrane transporter activity	GO:0015149	4		
hexose transport	GO:0008645	4		
homoserine metabolic process	GO:0009092	2		
**intrinsic to membrane**	**GO:0031224**	**30**	**13**	
mannose transmembrane transporter activity	GO:0015578	4		
membrane	GO:0016020	30	13	
membrane part	GO:0044425	30	13	
modified amino acid transmembrane transporter activity	GO:0072349	3		
monosaccharide binding	GO:0048029	2	2	
monosaccharide transmembrane transporter activity	GO:0015145	4		
monosaccharide transport	GO:0015749	4		
**multi-organism process**	**GO:0051704**	**7**	**5**	**1**
organic substance transport	GO:0071702	8		
peptide catabolic process	GO:0043171	2		
peptide metabolic process	GO:0006518	2		
**plasma membrane**	**GO:0005886**	**9**	**4**	
pseudohyphal growth	GO:0007124		4	3
regulation of anatomical structure size	GO:0090066		4	3
regulation of biological quality	GO:0065008			3
regulation of cell size	GO:0008361		4	3
regulation of cellular component size	GO:0032535		4	3
response to stimulus	GO:0050896		10	
response to toxin	GO:0009636	2	1	
sequence-specific DNA binding	GO:0043565			2
sugar binding	GO:0005529	2	2	
sugar transmembrane transporter activity	GO:0051119	4		
sulfur amino acid metabolic process	GO:0000096	3		
sulfur amino acid transmembrane transporter activity	GO:0000099	3		

GO Term	GO ID	S288c *MSS11*	Sigma *MSS11*	Sigma *mss11*
sulfur compound metabolic process	GO:0006790	5		
sulfur compound transport	GO:0072348	2		
transcription regulator activity	GO:0030528			2
