# Supplementary material for: Many Saccharomyces cerevisiae Cell Wall Protein Encoding Genes Are Coregulated by Mss11, but Cellular Adhesion Phenotypes Appear Only Flo Protein Dependent
Source: G3 (Bethesda). 2012 Jan 1;2(1):131–41. doi: 10.1534/g3.111.001644 (PMC3276193; doi:10.1534/g3.111.001644)
Supplement: Supporting Information [file supp_2.1.131_TableS7.pdf]

Table S7 GO enrichment analysis of genes significantly regulated in response to *FLO11* over-expression in ∑1278b (indicated as Σ1278b *FLO11*) using the online application FunSpec (p<0.0001; (Robinson *et al.* 2002). Shown is the number of genes identified to group in each category.

GO Term	GO ID	Sigma *FLO11+*
branched chain family amino acid catabolic process	GO:0009083	4
catalytic activity	GO:0003824	24
cellular aromatic compound metabolic process	GO:0006725	8
metabolic process	GO:0008152	21
organic acid metabolic process	GO:0006082	9
response to temperature stimulus	GO:0009266	5
transmembrane transport	GO:0055085	22
transporter activity	GO:0005215	13

REFERENCES

Bester, M. C., I. S. Pretorius and F. F. Bauer, 2006 The regulation of Saccharomyces cerevisiae *FLO* gene expression and Ca^{2+}-dependent flocculation by Flo8p and Mss11p. Curr Genet **49:** 375-383.

Brachmann, C. B., A. Davies, G. J. Cost, E. Caputo, J. Li *et al.*, 1998 Designer deletion strains derived from *Saccharomyces cerevisiae* S288C: a useful set of strains and plasmids for PCR-mediated gene disruption and other applications. Yeast **14:** 115-132.

Gagiano, M., D. Van Dyk, F. F. Bauer, M. G. Lambrechts and I. S. Pretorius, 1999a Divergent regulation of the evolutionarily closely related promoters of the *Saccharomyces cerevisiae STA2* and *MUC1* genes. J Bacteriol **181:** 6497-6508.

Gagiano, M., D. van Dyk, F. F. Bauer, M. G. Lambrechts and I. S. Pretorius, 1999b Msn1p/Mss10p, Mss11p and Muc1p/Flo11p are part of a signal transduction pathway downstream of Mep2p regulating invasive growth and pseudohyphal differentiation in *Saccharomyces cerevisiae*. Mol Microbiol **31:** 103-116.

Gietz, R. D., and A. Sugino, 1988 New yeast-*Escherichia coli* shuttle vectors constructed with in vitro mutagenized yeast genes lacking six-base pair restriction sites. Gene **74:** 527-534.

Robinson, M. D., J. Grigull, N. Mohammad and T. R. Hughes, 2002 FunSpec: a web-based cluster interpreter for yeast. BMC Bioinformatics **3:** 35.

van Dyk, D., I. S. Pretorius and F. F. Bauer, 2005 Mss11p is a central element of the regulatory network that controls *FLO11* expression and invasive growth in *Saccharomyces cerevisiae*. Genetics **169:** 91-106.

Verstrepen, K. J., A. Jansen, F. Lewitter and G. R. Fink, 2005 Intragenic tandem repeats generate functional variability. Nat Genet **37:** 986-990.
